# Plasmonic‐Quantum and Emitter's Finite‐Size Effects in Plasmon‐Emitter Coupling: A QHT–DFT Hybrid Approach

**DOI:** 10.1002/nap2.70044

**Published:** 2026-03-16

**Authors:** Tadele Orbula Otomalo, Gabriel Gil, Gonzalo Álvarez‐Pérez, Huatian Hu, Ferruccio Pisanello, Stefano Corni, Fabio Della Sala, Cristian Ciracì

**Affiliations:** ^1^ Center for Biomolecular Nanotechnologies Istituto Italiano di Tecnologia Arnesano Italy; ^2^ Università del Piemonte Orientale Alessandria Italy; ^3^ Università degli Studi di Padova Padova Italy; ^4^ Institute for Microelectronics and Microsystems (CNR‐IMM) Lecce Italy; ^5^ Neurophos Inc. Austin Texas USA

**Keywords:** DFT, electron tunneling, molecular spatial extent, nonlocality, *p*‐nitroaniline (PNA) molecule, plasmon‐emitter interactions, QHT

## Abstract

We implement a hybrid scheme that combines time‐dependent density functional theory (TDDFT) with quantum hydrodynamic theory (QHT). Using TDDFT, we capture the transition charge density of a *p*‐nitroaniline (PNA) molecule, which translates into a realistic field distribution used to excite the QHT‐treated plasmonic systems. This facilitates the investigation of plasmonic quantum effects and the emitter's spatial extent in plasmon‐emitter interactions. As a result, we compare the emission properties of the PNA and a point dipole (PD) in plasmonic dimer gaps. Significant discrepancies are observed in both the oscillator strengths of the emission rates and the spectral positions of the prominent modes in the PNA‐ and PD‐based models when the dimer gap size is smaller than approximately 3.2 nm. These discrepancies are attributable to the spatial extent of the PNA molecule. The gap at which quantum‐tunneling‐induced emission quenching begins is observed to be three times larger in the PNA‐based system than in the PD‐based one. We study these phenomena by progressively introducing the quantum effects, starting from the local response approximation, moving to the nonlocal Thomas–Fermi hydrodynamic theory, and ultimately extending to the full QHT.

## Graphical abstract

Plasmon‐emitter interactions in a dimer are studied using a hybrid approach: density functional theory captures the molecular transition density of the *p*‐nitroaniline molecule, whereas quantum hydrodynamic theory models the plasmonic system. Comparison with a point dipole shows significant deviations in emission rates and spectral positions for gaps below 3.2 nm, while tunneling‐induced quenching emerges near 1.64 nm, highlighting the interplay of molecular size and quantum effects.

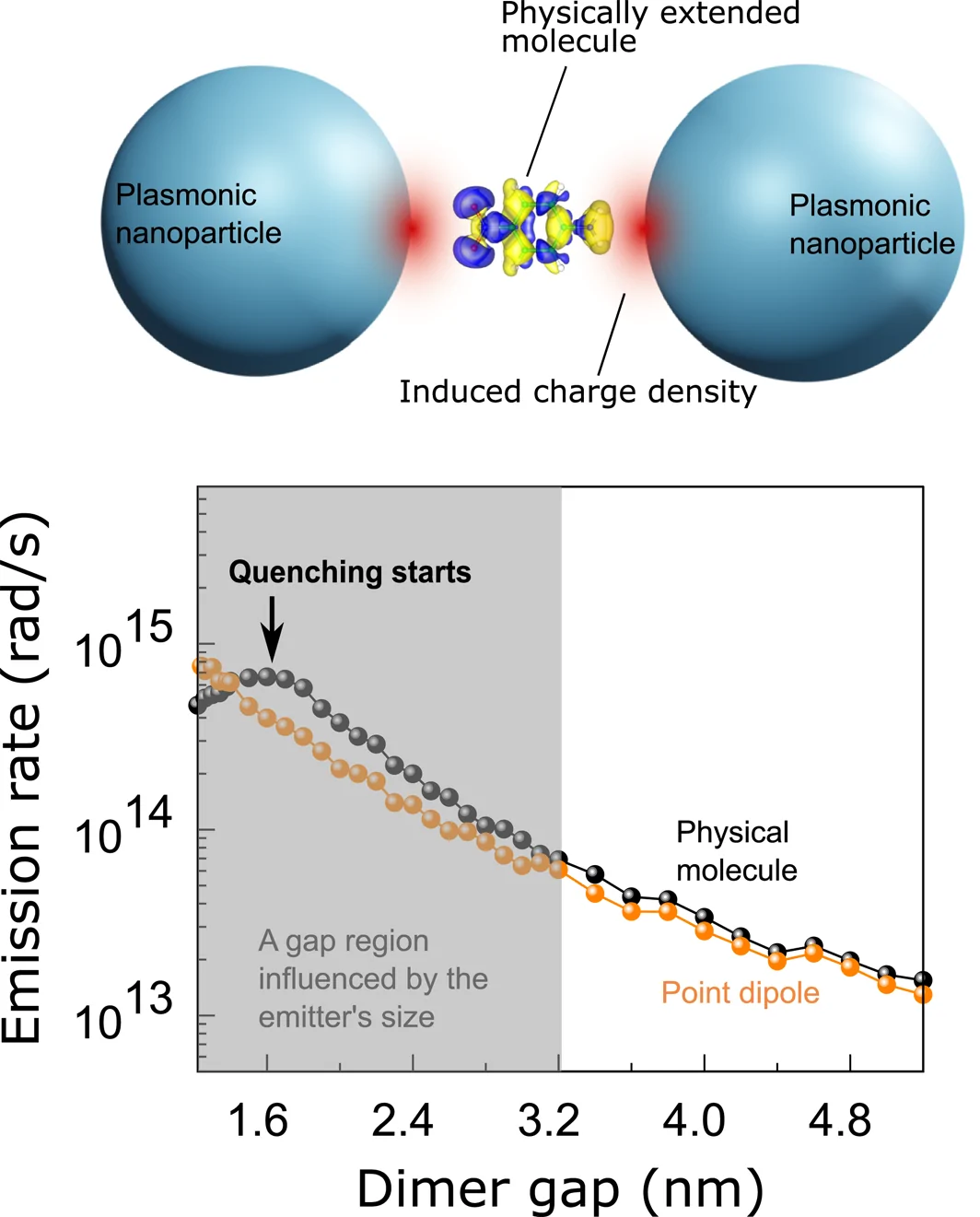

## 1 Introduction

The study of quantum‐emitter–plasmon coupling plays a key role in advancing nanophotonics, because of its multifaceted applications ranging from single‐photon sources [1, 2] to biomedical and environmental sensing [3, 4, 5], strong light–matter interactions [6, 7, 8] and nanophotonic devices [9, 10, 11]. When a quantum emitter, such as a molecule, a quantum dot, or an exciton, is placed near a plasmonic nanostructure, its emission dynamics can be drastically modified due to the strong confinement and enhancement of electromagnetic (EM) fields in the vicinity of the plasmonic system [12, 13]. This modification is often detected as an enhancement or suppression of the spontaneous emission rate of the emitter, a parameter closely related to the local density of states (LDOS), depending on its position near the plasmonic nanostructure [14]. The spatial variation of the LDOS and wavelength of the EM field are typically much larger than the physical size of an emitter; this has motivated the common simplification of modeling emitters as point dipole (PD) sources, known as the point dipole approximation (PDA). It can predict long‐range interactions between an emitter and a plasmonic system with reasonable accuracy [15, 16, 17]. However, as the separation distance between the emitter and the plasmonic nanostructure decreases, the finite spatial extent of the emitter becomes significant, necessitating a departure from this simplified model [18, 19, 20, 21]. This was effectively demonstrated by comparing the emission rates determined by the PDA with those calculated for molecular emitters, which are defined by their respective spatially dependent transition charge densities, where a significant discrepancy was observed at subnanometer emitter–plasmon separation distances [18, 20]. This inconsistency between point‐like and realistic emitters can be observed even at larger separations (up to 100 nm) when mesoscopic semiconductor quantum dots are involved [21]. Therefore, a precise modeling of emitter‐plasmon interactions under these conditions necessitates an emitter description that incorporates its spatial extent, beyond the limitations of the point‐like approximations. This includes semi‐classical methods, such as multipolar [19] and distributed dipole [22] models, and fully quantum approaches, such as quantum electrodynamics [23] and density functional theory (DFT) [20].

Furthermore, at the length scale in which the plasmonic systems can confine the strongest EM field, nonlocal, finite‐size, spill‐out, and quantum tunneling effects are expected, which are overlooked in the classical (local) models. These quantum mechanical phenomena, which arise mainly from quantum size effects and/or electron interactions, have been experimentally observed and are known to dramatically alter optical response of the plasmonic system [24, 25, 26, 27, 28, 29, 30, 31, 32, 33, 34]. To capture these effects, the most straightforward approach would be to implement a fully quantum mechanical method. However, this is computationally prohibitive and restricted to small systems or atomic clusters [35, 36, 37], leading to emergence of alternative approaches that solve the classical Maxwell equations, for the real world plasmonic systems which can reach sizes of up to several tens of nanometers, using the standard EM solvers while systematically embedding the quantum effects [38, 39, 40, 41, 42, 43, 44].

In this regard, the commonly employed semi‐classical models are Feibelman d‐parameter model [45] and QHT [41, 42]. The former offers the sought quantum correction using surface response parameters known as Feibelman d‐parameters, that can be obtained either from first‐principles [39] or analytical calculations [24]. It has been successfully utilized in plasmonics to capture microscopic surface effects such as spill‐out and charge smearing at the metal‐dielectric boundaries [24, 38, 39, 46]. QHT, on the other hand, characterizes both the surface and bulk electron gases. It is a fluid electron dynamics model that relies on bulk pressure and quantum gradient corrections, which allow it to capture a wide range of quantum effects in a self‐consistent way [40, 42, 43, 44]. What is particularly interesting about QHT is its adaptability for modeling both isolated plasmonic systems and those involving nanogaps [6, 44, 47, 48, 49, 50, 51].

Therefore, rather than completely relying on the computationally intensive ab initio techniques to account for the quantum plasmonic effects and the spatial extent of the emitters, an efficient strategy would be to adopt a hybrid approach, treating the emitters quantum mechanically, since their small size makes them more manageable, while modeling the plasmonic system using a semi‐classical electrodynamics, QHT for instance. In this study, we present such a hybrid scheme that combines TDDFT and QHT. We utilize the TDDFT to calculate the spatially resolved dipolar transition charge density of the PNA molecule, which then serves as a realistic excitation source for the QHT‐treated plasmonic systems. We specifically focus on exposing the consequences of neglecting the emitter's finite extent, with or without consideration of the plasmonic quantum effects. This will be done by comparing the emission properties of the TDDFT‐characterized molecule near plasmonic nanoparticle (NP) systems with those when a PD, whose moment is determined from the same molecule via the TDDFT simulation, is involved.

We begin by examining how the emitter's finite spatial extent influences its interaction with a fully classically described plasmonic system. Comparison of emission rates of such a system with those based on a PD is already well‐understood [18, 19, 20] and serves as a benchmark for our formalism. At large emitter‐plasmon distances, the molecular and corresponding PD emission rates converge, as anticipated, confirming that they characterize the same underlying emitter response and validating that implementation of the spatially resolved PNA molecule is reliable. However, at sub‐nanometer distances, large differences appear in both the weak and strong coupling regimes, highlighting the critical role of the emitter's physical extent in the near‐field interactions.

We then proceed to investigate the combined plasmonic quantum and emitter's extent effects by applying the hybrid model. To this end, we study the interaction between the emitters (PD and PNA) and a plasmonic dimer, a system known to exhibit rich quantum effects [6, 44, 47]. We analyze the PD‐ and PNA‐based emitter‐plasmon coupling by progressively introducing quantum effects into the system: starting with the fully classical description using the local response approximation (LRA), followed by the nonlocal Thomas–Fermi hydrodynamic theory (TFHT), which accounts for spatial field gradients within the plasmonic NPs, and finally employing the full QHT, which includes both nonlocality and electron quantum tunneling.

As the dimer‐gap narrows below g≈3.2 nm, substantial discrepancies emerge between the PD‐ and PNA‐based systems, both in the oscillator strength of the emission rates and spectral position of the prominent modes across all the employed models, LRA, TFHT, and QHT. Furthermore, the gap at which quantum‐tunneling‐induced emission quenching begins is observed to be three times larger in the PNA‐based system (g≈1.64 nm) than in the PD‐based one. This demonstrates that both the emitter size and the quantum corrections become critical in the small‐gap regimes. This combined effect is also found to critically affect the plasmon–emitter coupling strength. This technique of integrating fully quantum and semi‐classical electrodynamic approaches holds great potential for resolving some of the fundamental shortfalls in the study of emitter–plasmon interactions.

## 2 Results and Discussion

### 2.1 Quantum Hydrodynamic Theory

The foundation of hydrodynamic modeling is based on the collective behavior of free electrons, which can be treated as a fluid under applied EM fields (E and B). Two hydrodynamic variables characterize the electron many‐body dynamics of the system: the electron density n(r,t) and the velocity field v(r,t) [42]. The electronic system driven by the EM field obeys the hydrodynamic equation [42]:

(1) $$m_{e}\left(\frac{\partial }{\partial t}+v\cdot \nabla +\gamma \right)v=-e\left(E+v\times B\right)-\nabla \frac{\delta G\left[n\right]}{\delta n},$$

where $m_{e}$ and e are respectively the free electron mass and electron charge (absolute value), and γ is the phenomenological damping (collision) rate. The main terms in the equation are convection, v⋅∇v, the Lorentz force, and the gradient of the potential, $\left(\delta G/\delta n\right)$, where G[n] is the energy functional that accounts for the total internal energy of the free electron gas.

A hydrodynamic‐based light–matter interaction modeling requires coupling of Equation (1) with the EM wave equation. This is done by applying a continuity equation, $\dot{n}+\nabla \cdot \left(nv\right)=0$ together with the assumption $\dot{P}=J=-nev$, where P and J are polarization and current density, respectively and the dots on n and P denote time derivatives. The frequency‐domain linear response under the QHT framework in vacuum is then governed by Ref. [42]:

(2a) $$\nabla \times \nabla \times E-\frac{\omega^{2}}{c^{2}}E=\omega^{2}\mu_{0}P,$$

(2b) $$\frac{en_{0}}{m_{e}}\nabla {\left(\frac{\delta G}{\delta n}\right)}_{1}+\left(\omega^{2}+i\gamma \omega \right)P=-\varepsilon_{0}\omega_{p}^{2}E,$$

where $\varepsilon_{0}$ is vacuum permittivity, c is speed of light and $\omega_{p}\left(r\right)=\sqrt{e^{2}n_{0}\left(r\right)/\left(m_{e}\varepsilon_{0}\right)}$ is a space dependent, through ground state (equilibrium) electron density $n_{0}\left(r\right)$, plasma frequency. ${\left(\delta G/\delta n\right)}_{1}$ is first‐order functional derivative of G with respect to n in the perturbation theory scheme [42]. $n=n_{0}+n_{1}$ where $n_{1}=\left(1/e\right)\nabla \cdot P$ denotes first‐order linear perturbation of the charge density. If the first term involving the first‐order functional derivative is dropped, Equations (2a) and (2b) becomes just a local response approximation (LRA) model where the local dielectric response of the plasmonic system is considered.

In the present work, G[n] is considered to be the sum of interacting kinetic energy contribution, that is, the Thomas–Fermi $T_{\text{TF}}\left[n\right]$ and von Weizsäcker $T_{\text{vW}}$, and exchange–correlation potential energy $E_{\text{XC}}$. Hence the total energy functional is expressed as, $G\left[n\right]=T_{\text{TF}}\left[n\right]+T_{\text{vW}}\left[n\right]+E_{\text{XC}}\left[n\right]$. The explicit expressions for the first‐order functional derivative of these quantities can be found in Ref. [42]. If the last two terms in G[n], namely, $T_{\text{vW}}\left[n\right]$ and $E_{\text{XC}}\left[n\right]$, are dropped, and a constant equilibrium charge density, $n_{0}\left(r\right)=n_{0}$, is used with a hard wall boundary condition, then the model reduces to the Thomas‐Fermi hydrodynamic theory (TFHT) approximation. Assuming these conditions, $T_{\text{TF}}\left[n_{0}\right]$ simplifies to $\beta^{2}\nabla \left(\nabla \cdot P\right)$ where the parameter β is responsible for the electron pressure and can be expressed in terms of Fermi velocity, $v_{F}$, as $\beta^{2}=v_{F}^{2}/3$ [40].

The above QHT's set of equations (Equations 2a and 2b) is for solving a three‐dimensional problem. However, because of the axis‐symmetry of the geometries considered in this work, the simulations are carried out using a 2.5D approach. The 2.5D method is based on the representation of the vector fields in the equation using cylindrical harmonics and computing the response on a two‐dimensional cross‐section separately for each azimuthal harmonic, m, with m∈Z, granting a significant reduction of the computational cost [30, 52, 53, 54]. The 2.5D explicit form of Equations (2a) and (2b) is presented in the *Methods* section (Section 4.1).

### 2.2 Modeling the Emitters

The molecule studied in the present work is *p*‐nitroaniline (PNA), hereafter referred to as the PNA molecule, the molecule, or the physical molecule. It is planar and elongated along one axis, where both nitrogens of the molecule are located, that is the direction along the nitrogen–nitrogen (NN) bond between the amino (${\text{NH}}_{2}$) and nitro (${\text{NO}}_{2}$) functional groups. We assume that the molecule lies on the yz‐plane (x=0), with the NN axis aligned along z.

To model the physically extended PNA molecule, we follow a recently introduced *dummy*‐surface boundary element method (ds‐BEM) [55], which utilizes a *dummy* surface to formulate boundary integral equations, thereby mapping the molecular transition density associated with the first optically active excitation (i.e., GS→ES*) onto the *dummy* surface.

The modeling is carried out in a two‐step process. First, we perform a linear‐response time‐dependent density functional theory (LR‐TDDFT) calculation for the molecule at the B3LYP/6‐31G(d) level in a locally modified version of GAMESS‐US [56] (forked from v. 2014 R1). The GS→ES* transition density corresponding to this calculation is shown in Figure 1. Next, we use the ds‐BEM approach [55] to obtain an electrostatically equivalent representation of the GS→ES* transition density in terms of point charges located on the *dummy* surface. The molecular transition electrostatic potential values V are computed at the nodes of the ellipsoidal tessellation as follows:

(3) $$V=\sqrt{2}\sum_{ia}\left(X_{ia}+Y_{ia}\right)\langle i|\hat{V}|a\rangle ,$$

where $X_{ia}$ and $Y_{ia}$ are the excitation and de‐excitation amplitudes of the GS→ES* singlet transition, and $\langle i|\hat{V}|a\rangle$ are the matrix elements of the electrostatic potential operator in the molecular‐orbital basis evaluated at the tessellation nodes.

**FIGURE 1:**
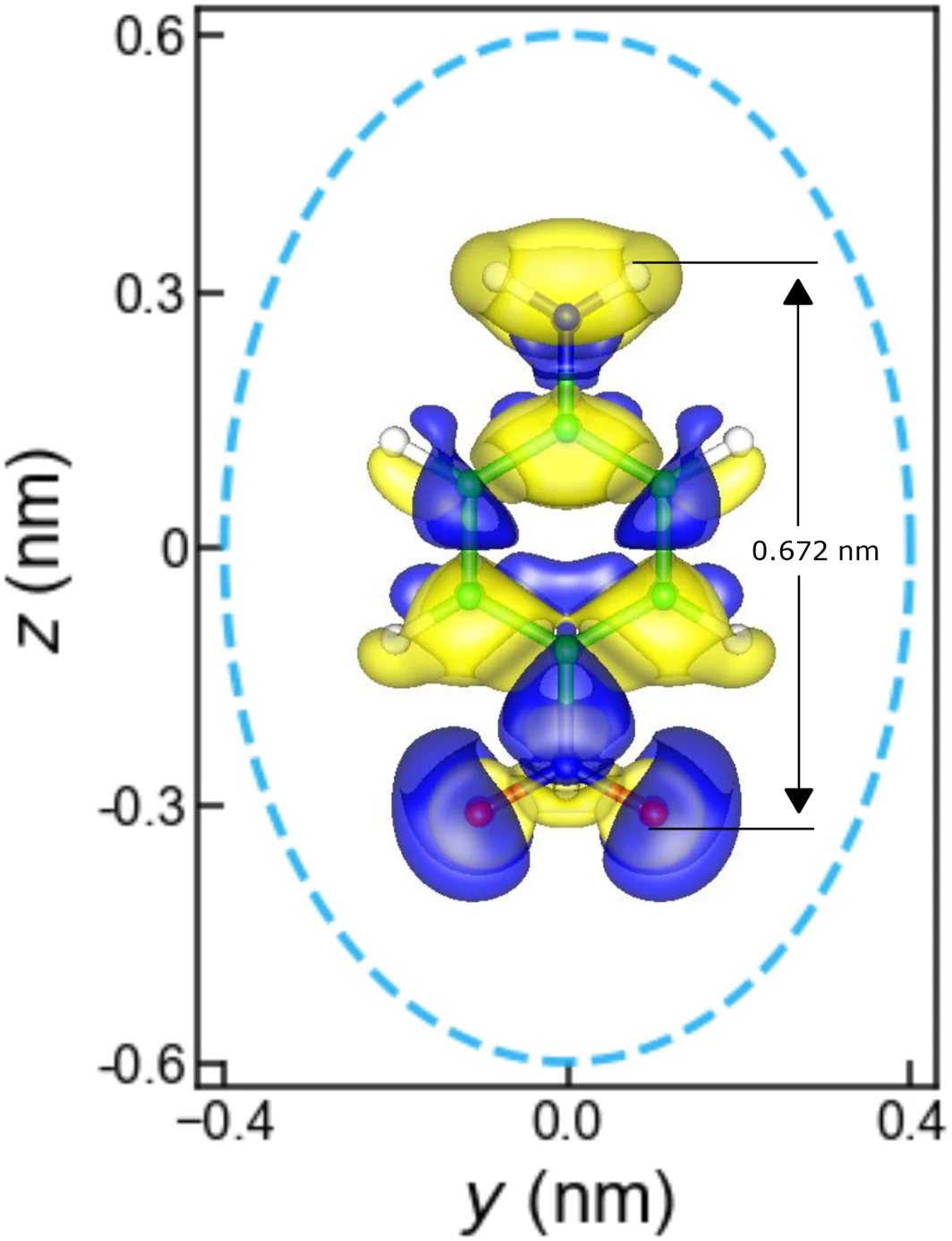
Two‐dimensional ball‐and‐stick representation of the PNA molecule in the *yz* plane, with total length $d_{\text{mol}}=0.672$ nm, shown together with the GS→ES* transition density contour plot obtained from LR‐TDDFT (isodensity = 0.008 au). An ellipsoidal *dummy* surface encloses the molecule; its corresponding two‐dimensional *yz* section is highlighted as an ellipse, shown as the dashed light‐blue curve.

As a result of its geometric features and chemical composition, the van der Waals volume of the molecule fits well within a prolate ellipsoid's *dummy* surface with semimajor and semiminor axes of 0.6 and 0.4 nm, respectively. The major axis is aligned along the NN axis of the molecule, and the molecule has a size of $d_{\text{mol}}=0.672$ nm along this axis (see Figure 1).

Finally, we employ a Gmsh [57] discretization of this surface into $N_{\text{tess}}=2,774$ triangular tesserae to construct an electrostatically equivalent surface representation of the PNA transition density corresponding to its first optically active excitation, GS→ES*.

The TDDFT transition potential values on the ellipsoid are imposed as boundary conditions to solve the inverse electrostatic problem, yielding the distribution of point charges supported on the same surface. The layer potential generated by these charges exactly reproduces the TDDFT transition potential everywhere outside the ellipsoidal surface. In practice, the set of charges q at the characteristic points of the ellipsoidal tessellation is obtained as follows:

(4) $$q=S^{-1}V,$$

where S is the Calderon BEM $N_{\text{tess}}\times N_{\text{tess}}$ matrix containing Coulomb interactions between tesserae on the *dummy* surface. In the broader context of the QHT–DFT hybrid modeling of the plasmon–emitter interactions, this procedure is valid because the plasmonic NP lies entirely in the region external to the ellipsoidal *dummy* surface.

In Figure 2, we plot the xz and yz projections of the charge distribution (Figure 2A) and the resulting electric field distribution in air (Figure 2B), computed using a Finite Element Method (FEM), of the molecule. The GS→ES* transition density (with excitation energy 4.05 eV) has a strongly polar character, featuring large charge separation along the molecular axis in its *dummy*‐surface representation. The orientation and magnitude of the transition electric dipole moment μ as derived from ds‐BEM is in quantitative agreement with the direct computation via LR‐TDDFT, yielding the Cartesian components $\mu_{x}=-1.3946\times 10^{-5}$ D, $\mu_{y}=4.5100\times 10^{-5}$ D, and $\mu_{z}=4.6333$ D, where 1 D (debye) is $\approx 3.3356\times 10^{-30}$ C⋅m. Such a vector represents a hypothetical PD residing at the geometrical center of the prolate ellipsoid. The charge cloud defined by q and the dipole moment μ are the necessary inputs for modeling the interaction between the quantum emitter and the plasmonic system.

**FIGURE 2:**
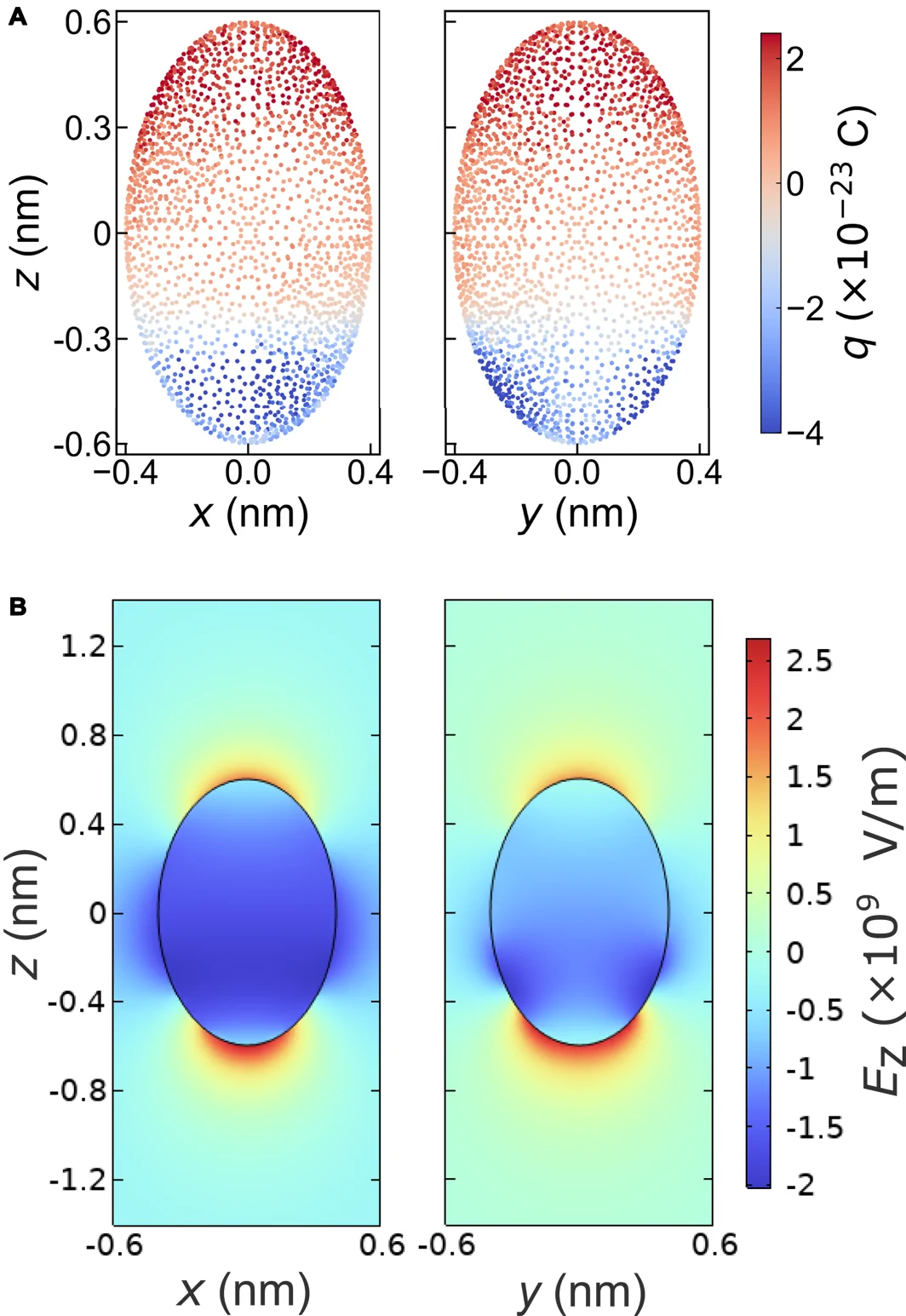
Charge distribution and resulting electric field of the PNA molecule, illustrating its dipolar nature. (A) zx (left panel) and zy (right panel) projections of the charge distribution q over a prolate ellipsoid with semiminor and semimajor axes of 0.6 and 0.4 nm, respectively. This distribution reproduces the transition potential associated with the GS→ES* excitation of PNA outside the ellipsoid, thus serving as an equivalent representation of the molecule's transition density. (B) The z‐component of the electric field $\left(E_{z}\right)$ emitted by the PNA molecule in air, computed using FEM. Left panel: shows the spatial distribution of $E_{z}$ in the zx‐plane, capturing the variation of the field along the z‐ and x‐direction at y=0. Right panel: displays the distribution in the zy‐plane, showing the variation along the z‐ and y‐direction at a fixed x=0.

For the PNA molecule‐plasmonic interactions, a spatially distributed electric field, generated by the dipolar surface charge distribution of the molecule, is used to excite the plasmonic systems. For this, the electric field of the charge distribution on the surface of the ellipsoid is computed from Coulomb's law. We calculate the electrostatic field at the position of each point charge by summing the Coulombic contributions from all other charges, excluding self‐interaction. For a discrete collection of point charges, the electric field at a point r=(x,y,z) due to all other charges $q_{j}$ located at $r_{j}=\left(x_{j},y_{j},z_{j}\right)$ is given by the following equation:

(5) $$E\left(r\right)=\sum_{j}\frac{q_{j}}{4\pi \varepsilon_{0}}\frac{\left(r-r_{j}\right)}{|r-r_{j}{|}^{3}},$$

where $r-r_{j}$ is the vector pointing from the source point j to the observation point, and $|r-r_{j}|=\sqrt{{\left(x-x_{j}\right)}^{2}+{\left(y-y_{j}\right)}^{2}+{\left(z-z_{j}\right)}^{2}}$ is the corresponding scalar distance. Here, $\varepsilon_{0}$ is the vacuum permittivity.

For implementation in a 3D numerical simulation, the three‐dimensional components of the static electric field, $E_{x}$, $E_{y}$, and $E_{z}$, evaluated using Equation (5), can be defined on the surface of a prolate ellipsoid representing the PNA molecule, as been determined by TDDFT modeling. Consequently, the field outside the ellipsoid (the molecule) is computed. Figure 2B demonstrates this, showing the z‐component of the electric field distribution emitted by the molecule in air, evaluated via FEM in COMSOL Multiphysics [58].

However, in the present work, we implement a 2.5D simulation method, which requires significantly fewer computational resources than the full 3D approach. For this purpose, once the three‐dimensional components of the molecular field are determined using Equation (5), they are subsequently transformed into a representation compatible with the 2.5D axisymmetric modeling framework (for details, see Section 4.2 in *Methods*). This approach yields the same electric‐field distribution as in Figure 2B when revolved into three dimensions, while being computationally more efficient. The resulting field is imposed on the boundary of the molecular geometry in the FEM simulations as a source field emitted by the molecule. Positioned at a prescribed emitter location, the molecule thus acts as a source that excites the plasmonic system in the LRA, TFHT, and QHT models.

The PD is also modeled using a 2.5D formulation that relies on a Fourier expansion of the Dirac delta function associated with the dipole's current [53]. For an oscillating PD of moment μ and location $r=r_{0}$, its current density is $j=-i\omega \delta^{3}\left(r-r_{0}\right)\mu$. Thus, the wave equation analogous to the general one (Equation 2a), but for a PD source, can be expressed as follows:

(6) $$\nabla \times \nabla \times E-\frac{\omega^{2}}{c^{2}}E=\omega^{2}\mu_{0}\delta^{3}\left(r-r_{0}\right)\mu .$$

This approach is especially effective for modeling PDs of arbitrary position and polarization [53]. Because the PD considered here has a three‐component moment, as computed from the first‐principles application, it is the appropriate one as it accepts these components, $\mu_{x}$, $\mu_{y}$, and $\mu_{z}$, as input for defining the source term. The detail, including the explicit 2.5D formulation of Equation (6) can be found in Ref. [53].

### 2.3 Analyzing the Emitter‐Driven Emitter‐Plasmon Coupling

This subsection details the methodologies employed to analyze the emission characteristics of emitters in the presence of plasmonic systems. Throughout the following, we consider systems in which plasmonic nanostructures are excited by the field emitted by a dipole emitter, either a PNA molecule or a PD [6, 47]. This simplified approach is appropriate, as our objective is to investigate generic emitter–plasmon interactions, with a focus on the influence of the emitter's spatial extent and plasmonic quantum effects.

For the PNA molecule, the realistic molecular transition density corresponding to the GS→ES excitation at 4.05 eV (Section 2.2) is used to represent the emitter across the range of emission and dipole frequencies considered in this work. To avoid confusion, both the dipole frequencies $\hbar \omega_{0}$ and emission frequencies ℏω are chosen to lie below the excitation energy of the PNA molecule. Accordingly, we perform our analysis within the spectral range of 2–4 eV throughout this paper.

In such a setup, where a quantum emitter is placed near a plasmonic structure, the emission rates can be determined from the calculated electromagnetic fields by integrating the dissipated and scattered power [59]. The emission rates are of particular interest here as the plasmonic system can significantly influence them [59]. The non‐radiative decay rate, $\gamma_{nr}$, is found by integrating the absorbed power over the plasmonic volume, whereas the radiative decay rate, $\gamma_{r}$, is calculated from the power flux through a surface enclosing the emitter‐NP system (see Supporting Information S1: Note S1).

To analyze the coupling strength between the emitters and the plasmonic systems, we use the emission spectrum of a dipole emitter in the presence of a plasmonic system, which can be described as follows [6, 38, 60, 61]:

(7) $$S\left(\omega \right)=\frac{\hbar \omega }{2\pi }q\left(\omega \right)\gamma_{\text{tot}}\left(\omega \right)S^{\prime }\left(\omega \right).$$

where ω is the emitted frequency, q(ω) is the quantum yield, and $\gamma_{\mathrm{tot}}=\gamma_{nr}+\gamma_{r}$ is the total emission rate. Specifically for our system, this represents a power spectrum emitted into far field, averaged over all solid angles. Here, $S^{\prime }$ is a normalized dipole spectral function, defined as follows [6, 60, 61]:

(8) $$S^{\prime }\left(\omega \right)={\left|\frac{1}{i\left[\omega_{0}-\omega -\delta \omega \left(\omega \right)\right]+\frac{1}{2}\gamma_{\text{tot}}}\right|}^{2},$$

where $\omega_{0}$ is the dipole frequency of the emitter. The term δω(ω) is a frequency shift, also called the Lamb shift, induced by the plasmonic system near the emitter. The factor $S^{\prime }\left(\omega \right)$ is known as a dipole (polarization) spectrum [60], which holds most of the valuable information about the emission process. It represents the overall number of emitted photons, including those which do not reach the far field [6]. This approach is particularly well‐suited for systems with multiple emission peaks (as in our case), as it captures all the strong coupling features associated with these modes. For this, the Lamb shift, δ(ω), is determined by exploiting the relationship between the total emission rate, $\gamma_{\text{tot}}$, and Green's function of the system (see Supporting Information S1: Equation SI‐5 in Note S1).

### 2.4 Local Response Approximation (LRA)

The spatial extent of emitters is known to play a significant role at sub‐nanometer emitter‐plasmonic separation distances, even when the plasmonic system is described using purely classical models [18, 19, 20]. Therefore, before implementing the QHT, we first investigate this effect using LRA in both weak‐ and strong‐coupling regimes. Accordingly, we model the interaction between the emitters, a PD and a PNA molecule, and a plasmonic system comprising a 40 nm spherical gold NP (AuNP) in air. In particular, we examine the interaction between the emitters and both the single AuNP and its dimer. The experimental dielectric function data of gold, as reported in Ref. [62], is used to describe the AuNP. The simulation is configured to ensure strong interaction between the emitters and the plasmonic system. For this purpose, the dominant dipole moment, $\mu_{z}$, of the PD is aligned perpendicular to the AuNP surface in the single AuNP case, and along the dimer axis in the case of the dimer. For the PNA molecule, its long axis (the major axis) is oriented normal to the AuNP surface for the AuNP‐molecule configuration, and made to align with the dimer axis in the dimer configuration.

Figure 3 shows emission rates of the PD and PNA molecule as a function of their separation distance, $d-d_{0}$, from the single AuNP, evaluated near the surface plasmon resonance (SPR) frequency, ℏω=2.46 eV. For the molecule, d is defined as the distance from its geometric center to the NP surface. Similarly, for the PD, d denotes the distance from its location to the AuNP surface. Here, $d_{0}$ is half of the molecular length, $d_{0}=\frac{1}{2}d_{\text{mol}}$; therefore, $d-d_{0}$ represents the distance between the molecule (measured from its atoms) and the AuNP surface.

**FIGURE 3:**
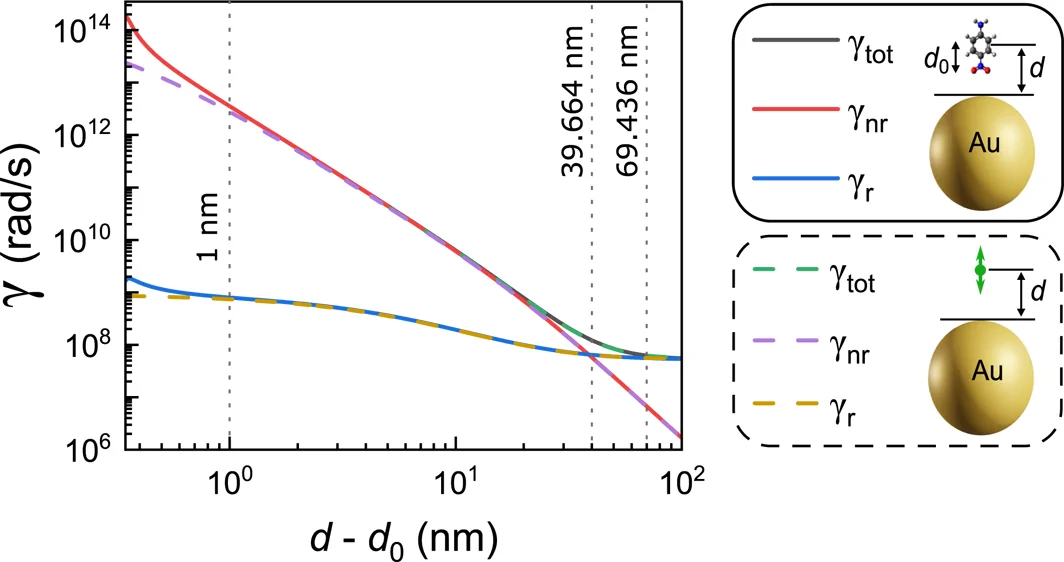
Comparison of emission rates, radiative $\left(\gamma_{r}\right)$, non‐radiative $\left(\gamma_{nr}\right)$, and total $\left(\gamma_{\mathrm{tot}}\right)$, as a function of the emitter‐AuNP separation distance $d-d_{0}$, evaluated at the SPR frequency, ℏω=2.46 eV, where $d_{0}=0.336$ nm corresponds to half the molecular length, $d_{0}=\frac{1}{2}d_{\text{mol}}$. Note that both axes of the plot use logarithmic scaling. d is measured from the emitters' center to the surface of the AuNP (radius 20 nm). The vertical dotted lines indicate $d-d_{0}=1$, 39.664, and 69.436 nm, marking the onset of the PNA finite‐size effect, the crossover between $\gamma_{nr}$ and $\gamma_{r}$ dominance, and the transition from $\gamma_{nr}$‐ to $\gamma_{r}$‐dominated behavior, respectively. The legend is shown in the panels on the right‐hand side. Solid lines represent the interaction between the PNA molecule and AuNP (top right panel), whereas dashed lines correspond to the PD–AuNP interaction (bottom right panel).

Both the PD and molecule display identical d‐dependent behavior. With decreasing d, $\gamma_{\text{tot}}$ increases due to the dominant non‐radiative energy transfer to the AuNP, marked by the coincidence between $\gamma_{\text{tot}}$ and $\gamma_{\text{nr}}$. There is a crossover around $d-d_{0}\approx 39.664$ nm (d≈40 nm), where the dominant energy transfer mechanism shifts from non‐radiative to radiative. This creates a transition region where $\gamma_{\text{tot}}$ is different from both $\gamma_{\text{nr}}$ and $\gamma_{r}$. Beyond $d-d_{0}\approx 69.436$ nm (d≈65.772 nm), $\gamma_{\text{tot}}$ aligns closely with $\gamma_{r}$, indicating radiative dominance (see the vertical dotted lines in Figure 3). Both systems follow the expected behavior of a dipolar emitter near a plasmonic surface [63]. Consequently, the overall quantitative agreement between the emission rates of the molecule and the PD confirms that the TDDFT‐calculated transition density and its corresponding dipole moment effectively represent the same underlying emitter behavior. Therefore, comparing the molecule‐ and PD‐based approaches is justified to assess any discrepancies between them.

However, a more careful examination of the sub‐nanometer range of $d-d_{0}$ reveals a clear deviation in the emission rates of the PNA molecule from those of the PD, with the molecule having larger emission rates. As noted in Section 2.2, transition density of the PNA molecule is strongly dipolar. Therefore, the main difference between the PD and full transition density models arises from the absence of spatial extension of the dipole moment in the former, rather than from possible contributions of higher‐order modes implicitly included in the latter. Overall, this deviation underscores the importance of accounting for the finite spatial extent of the molecule and its interaction with the near‐field of the AuNP.

Moreover, in this sub‐nanometer range, the molecule‐AuNP‐based system exhibits a redshift of the LSPR mode in the emission spectra with decreasing d, indicating strong coupling, an effect not observed in the spectra of the PD‐based system (see the d‐dependent spectra in Supporting Information S1: Figure S1). To verify this, we calculate the dipole spectrum $S^{\prime }\left(\omega \right)$ for both systems at d=0.61 nm ($d-d_{0}=0.274$ nm), using Equation (8); see Figure 4A. Although the nominal distance d=0.61 nm is identical for both systems, the metal–emitter separation $\left(d-d_{0}\right)$ for the extended molecule differs from that of the PD, with the extended molecule exhibiting a smaller molecule–metal separation. This leads to stronger near‐field interaction for the spatially extended emitter, producing a clear Rabi‐oscillation‐induced splitting near resonance (left panel of Figure 4A), whereas the PD‐based system (right panel of Figure 4A) does not exhibit splitting under the same nominal distance.

**FIGURE 4:**
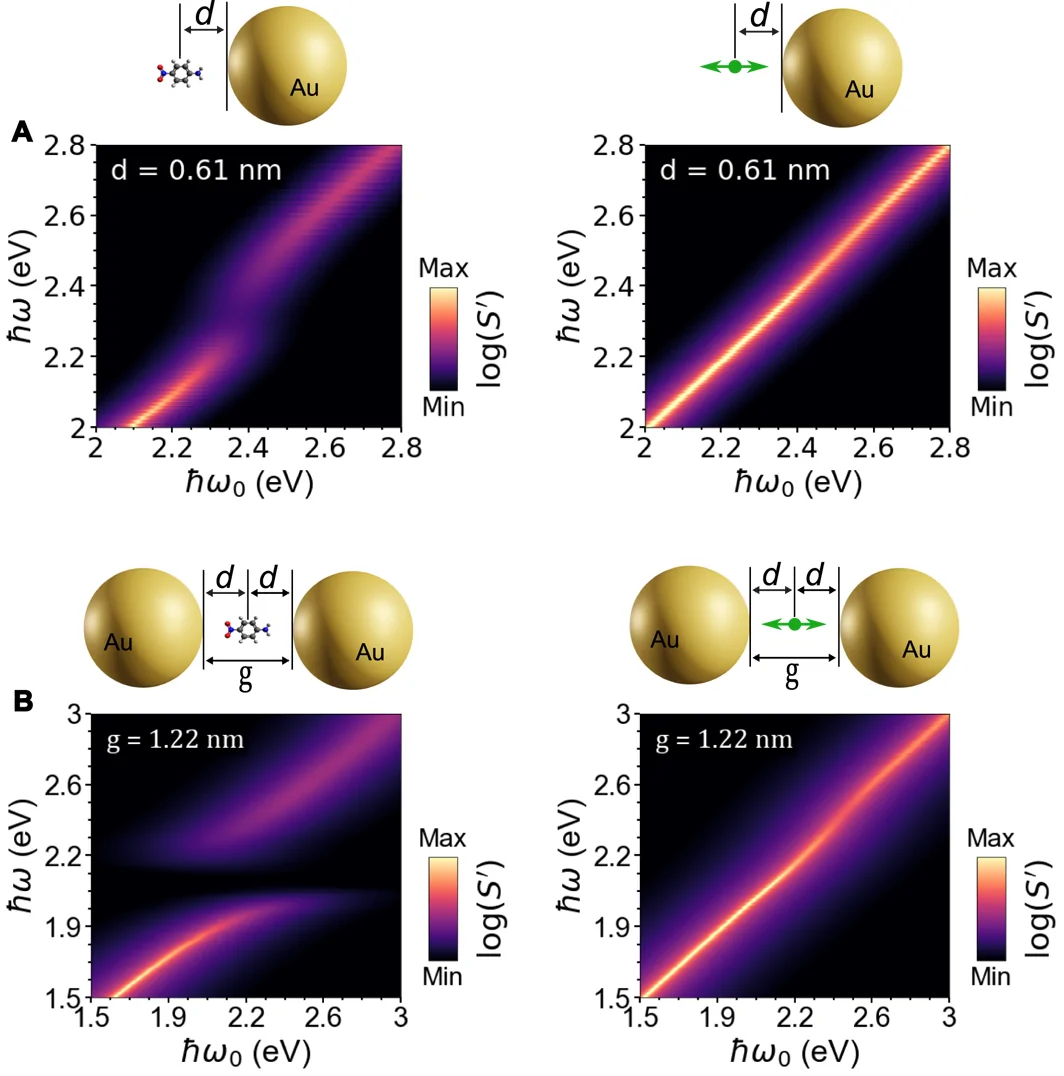
Comparison of emitter‐plasmon coupling strengths. Illustrations above each panel depict the corresponding emitter–plasmon configuration. (A) Dipole spectra, $S^{\prime }$, of the molecule (left panel) and PD (right panel), placed at a distance d=0.61 nm ($d-d_{0}=0.274$ nm with $d_{0}=\frac{1}{2}d_{\text{mol}}$ and $d_{\text{mol}}=0.672$ nm is the PNA molecular size) from a single AuNP of radius 20 nm, plotted as a function of the dipole frequency, $\hbar \omega_{0}$. (B) Same as in (A), but with emitters placed at the center of a plasmonic dimer gap of g=1.22 nm (molecule‐metal separation; $\left(g-d_{\text{mol}}\right)/2=0.274$ nm). The dimer consists of two identical AuNPs, each with a radius of 20 nm. The left panel shows the PNA‐based system, and the right panel shows the dimer–PD system.

We then scale up our plasmonic geometry to a dimer configuration to create more favorable conditions for strong coupling, leveraging its ability to confine significantly stronger electromagnetic fields in the gap due to the coupling of surface plasmon modes between the constituent AuNPs. Here, the emitters are located at the center of the dimer‐gap, and the size of the gap is defined as g=2d with d being the distance between the center of the gap and the surface of either AuNP (see the schemes on top of each panel in Figure 4B). For the dimer–molecule system, center of the molecule (its geometric center) coincides with the gap center (the scheme on top of the left panel in Figure 4B), whereas in the PD–dimer system, the dipole sits at the center of the gap (the scheme on top of the right panel in Figure 4B). Thus, d is consistently defined as d=g/2 in both systems. Hence, given the molecular length $d_{\text{mol}}=0.672$ nm (see Section 2.2); thus, $\left(g-d_{\text{mol}}\right)/2$ corresponds to the separation between the molecule and each adjacent AuNP.

The g‐dependent emission spectra of these systems are depicted in Supporting Information S1: Figure S2. Here, we analyze their dipole spectra at a dimer gap of g=1.22 nm (molecule–metal separation; $\left(g-d_{\text{mol}}\right)/2=0.274$ nm) to evaluate the strength of the coupling regime. The molecule‐dimer system shows much (left panel of Figure 4B), indicating a stronger emitter–plasmon interaction than observed in the molecule–AuNP case (the left panel in Figure 4A). Meanwhile, the PD–dimer system does not display a clear signature of strong coupling (right panel of Figure 4B).

In general, these differences between the molecule‐based and PD‐based systems at very short emitter‐plasmon separation distances, both in the weak and strong coupling regimes, show how the emitter's physical extent matters in this interaction range. In the following section, we study this effect by adding more layers of complexity to the description of the plasmonic systems by adding quantum effects.

### 2.5 The DFT–QHT Hybrid Model

In the preceding subsection, we have benchmarked the spatially extended molecule against the PD, observing consistent behavior at larger emitter–plasmon separations. This consistency supports the validity of using both models for further comparison. Here, we take a step further by integrating DFT and QHT to develop a DFT–QHT hybrid model. We conduct a similar comparison to assess the impact of both the emitter's spatial extent and plasmonic quantum effects on the interaction between the emitter and the plasmonic system. Implementation of the QHT and DFT techniques has already been covered in Section 2. Section 2.1 provides a general concept on the QHT formalism, whereas Section 2.2 introduces the usage of LR‐TDDFT to model the PNA molecule.

Sodium (Na) is chosen for this study because it has no significant interband excitations and is well‐suited for the QHT, which is tailored to model free‐electron plasmonics. Moreover, we focus our study on dimer geometries, as their narrow interparticle gaps provide a rich platform for exploring quantum phenomena. They exhibit all the quantum effects simultaneously [64], including spill‐out/tunneling and nonlocality, which are the conditions best suited for the QHT modeling [44, 47, 51].

The system under investigation is a Drude‐like Na nanoparticle (NaNP), characterized by the following parameters: plasma frequency $\omega_{p}=5.89$ eV and collision rate γ=0.234 eV (see Equation 2b) [44]. We explore a homodimer made of NaNPs of radius R arranged along the z‐axis, with a surface‐to‐surface separation g. Specifically, NaNP‐1 (the lower NP) is centered at z=−(d+R) and NaNP‐2 (the upper NP) is centered at z=(d+R), where d=g/2. The spatially dependent initial equilibrium electron density for the QHT is then modeled using the following expression [44, 47, 51],

(9) $$n_{0}\left(\rho ,z\right)=\frac{f_{0}}{1+e^{\kappa \left[r_{1}\left(\rho ,z\right)-R\right]}}+\frac{f_{0}}{1+e^{\kappa \left[r_{2}\left(\rho ,z\right)-R\right]}},$$

where $r_{1}\left(\rho ,z\right)=\sqrt{\rho^{2}+{\left(z+d+R\right)}^{2}}$ and $r_{2}\left(\rho ,z\right)=\sqrt{\rho^{2}+{\left(z-d-R\right)}^{2}}$ are respectively radial distances from the geometric centers of NaNP‐1 and NaNP‐2 to an observation point (ρ,z). $\kappa =1.05/a_{0}$, where $a_{0}$ is the Bohr radius, is a parameter obtained by fitting the asymptotic decay of Kohn–Sham densities [42]. $f_{0}$ is a normalization factor ensuring that the numerically calculated equilibrium electron density, via Equation (9), matches the uniform positive background charge density, defined according to the Jellium approximation, $n^{+}={\left(4/3\pi r_{s}^{3}\right)}^{-1}/a_{0}^{3}$, where $r_{s}=4$ a.u. is Wigner–Seitz radius of Na. Table 1 summarizes the parameters used to describe the NaNP.

**TABLE 1:** Material parameters for the Na‐jellium‐QHT model.

Parameter	Symbol	Value
Bulk plasmon frequency	$\omega_{p}$	5.89eV
Drude damping rate	γ	0.234eV
Wigner–Seitz radius	$r_{s}$	$4\;a_{0}$
Background charge density	$n^{+}$	${\left(\frac{4}{3}\pi r_{s}^{3}\right)}^{-1}/a_{0}^{3}$

We consider a dimer composed of NaNPs with R=10 nm. As previously demonstrated by Baghramyan and Ciracì [47], the spectral range in which such a dimer exhibits a significant emission efficiency spans from 2 to 4 eV. Accordingly, we restrict our analysis to this spectral range.

In Figure 5 we compare the spectral‐dependent total emission rates, $\gamma_{\mathrm{tot}}$, for the molecule‐ and PD‐based systems for two dimer‐gaps, g=1.3 nm (PNA‐NaNP separation; $\left(g-d_{\text{mol}}\right)/2=0.314$ nm) and g=3.2 nm (PNA–NaNP separation of 1.264 nm) as calculated by the different approaches, LRA (leftmost column), TFHT (middle column), and QHT (right column). The spectra are characterized by a distinct dipolar mode at lower photon energies and several higher‐order modes, depending on the employed simulation method, that is LRA, TFHT, and QHT [42]. The top panels show the spectra calculated at g=3.2 nm, whereas the bottom panels correspond to those calculated at g=1.3 nm.

**FIGURE 5:**
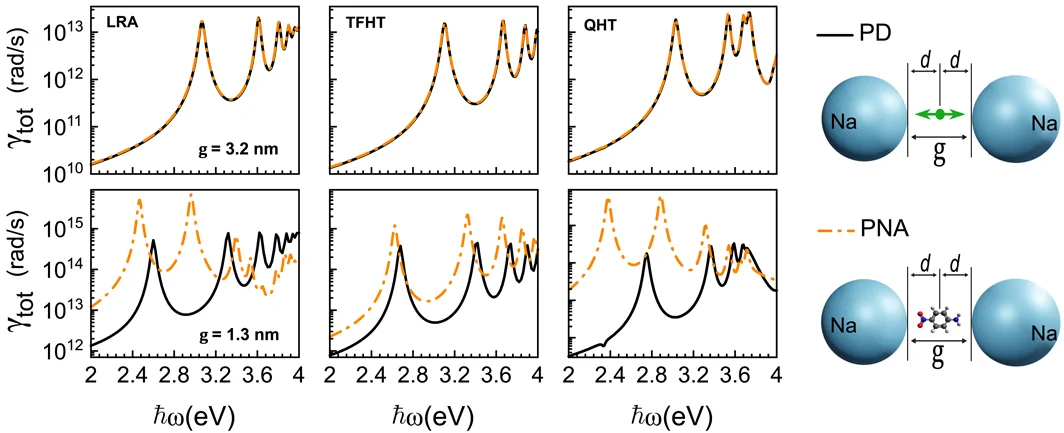
Emission spectra of dipolar emitters placed in NaNP dimer‐gap, g. As indicated by the legend on the right‐hand side, which includes illustrations of the configurations under consideration, the black solid lines represent the PD–dimer system, while the orange dash‐dotted curves correspond to the molecule‐based system. The plots show the total emission rates, $\gamma_{\mathrm{tot}}$, in log scale, for two sample dimer‐gaps: g=1.3 nm (PNA–NaNP separation; $\left(g-d_{\text{mol}}\right)/2=0.314$ nm) and g=3.2 nm (PNA–NaNP separation of 1.264 nm). Each column corresponds to one of the three modeling approaches used in this work: from left to right, LRA, TFHT, and QHT. The top panels show the spectra evaluated at g=3.2 nm, while the bottom panels correspond to g=1.3 nm.

When evaluated at the largest dimer gap considered here (g=3.2 nm), all the three computational methods (LRA, TFHT, and QHT) produce emission spectra that overlap perfectly for the PD‐based (black solid curves) and PNA‐based (orange dash‐dotted curves) models, see the top panels in Figure 5. However, at the smallest gap (g=1.3 nm), some of the emission features begin to change significantly between the two systems (PNA‐ and PD‐based), see the lower panels in 5. All modeling approaches reveal differences between the two systems in both the oscillator strength of the modes and their spectral positions. Although the differences in the spectral features are relatively minor in the TFHT‐treated system, they become considerably more pronounced in the QHT‐ and LRA‐treated systems. At such a small gap, the LRA predicts a strongly confined EM field, whereas the electron spill‐out induced effects begin to get introduced in the QHT [47]. This leads to a difference in the interactions of the plasmonic dimer with the spatially extended molecule and the PD, as will be detailed in the following discussion.

Let us now examine the spectra in more detail. For this, in Figure 6 we plot the spectral‐ and g‐dependent (using the PNA–NaNP separation parameter; $\left(g-d_{\text{mol}}\right)/2$) map of the non‐radiative (upper panels) and radiative (lower panels) emission rates for both the molecule‐based (Figure 6A) and PD‐based (Figure 6B) systems. The behavior at the smaller dimer‐gaps seems to be more apparent now. Roughly speaking, the spectra shown in Figure 6B resemble a portion of Figure 6A that remains after truncating it at around $\left(g-d_{\text{mol}}\right)/2\approx 0.364$ nm (g≈1.4 nm), without the information corresponding to the smaller gaps. In the smaller gap range, $\left(g-d_{\text{mol}}\right)/2≲0.364$ nm, and in the spectral region of ℏω≳3 eV in Figure 6A, the LRA‐treated molecule‐based system seems to be highly affected by spectral mode overlap. This mainly happens in the LRA because: (I) it predicts a strongly confined EM field compared to the other models (TFHT and QHT) [47], (II) the spatially extended emitter, as demonstrated using spatial overlap integrals, enables substantial coupling to higher‐order plasmonic modes, beyond what is typically captured by the PDA [18], and (III) these higher‐order modes are particularly susceptible to interference effects in the near‐field regime, especially at small gap sizes [65]. These factors account for the mode overlap observed in the LRA‐treated molecule‐based system and, consequently, the significant differences in the spectral range of the higher‐order modes relative to its PD‐based counterpart as shown in the bottom panel of the first column in Figure 5 for g=1.3 nm.

**FIGURE 6:**
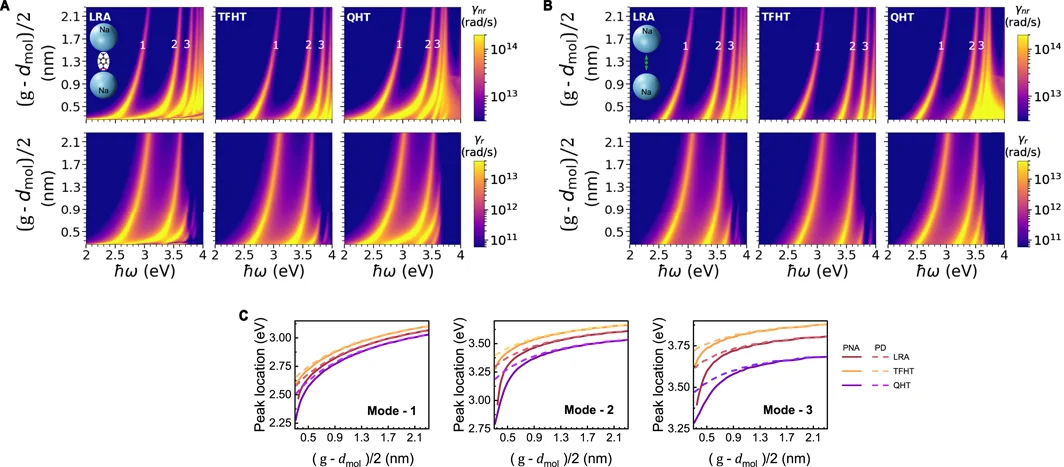
Gap‐resolved emission landscapes of the dipolar emitters in NaNP‐based dimers, with gap sizes, g, ranging from $\left(g-d_{\text{mol}}\right)/2=0.274$ nm (g=1.22 nm) to $\left(g-d_{\text{mol}}\right)/2=2.264$ nm (g=5.2 nm). (A) The color maps correspond to the non‐radiative, $\gamma_{nr}$, (upper panels) and radiative, $\gamma_{r}$, (lower panels) decay rates of the molecule‐dimer system calculated using LRA (leftmost column), TFHT (middle column), and QHT (last column). The inset in the top panel of the leftmost column illustrates the configuration in question: a PNA molecule placed at the very center of the NaNP dimer. (B) The same as in (A) but for a PD. (C) Comparison of the dimer‐gap‐dependent spectral peak positions for the molecule‐ and PD‐based systems. The three panels show the peak positions of the first three resonant modes, labeled 1, 2, and 3 in the upper panels of (A) and (B). These spectral positions correspond to maxima of the total emission rate, $\gamma_{\mathrm{tot}}$, for both systems as indicated in the legend (on the right side of the panels). The solid lines are for the molecule‐based system while the dashed lines are for the PD‐based one. The left, middle, and right panels respectively depict the g‐dependent spectral location of the first, second, and third modes.

Turning to the quantitative features of the spectral data, Figure 6C shows the dimer‐gap‐dependent spectral peak positions, corresponding to the maxima of the total emission rate, $\gamma_{\mathrm{tot}}$, for both the molecule‐based (solid lines) and PD‐based (dashed lines) systems, across the LRA, TFHT, and QHT methods. Here we focus on the mostly resolved peaks for all the gaps considered here, labeled as 1, 2, and 3 in the upper panels of Figure 6A,B. All the emission modes undergo a redshift when the dimer‐gaps (emitter‐metal separations) get narrower due to the interaction between the plasmonic NPs forming the dimer. Locations of the corresponding modes of the two systems appear to be consistent for larger metal‐emitter separations, but from $\left(g-d_{\text{mol}}\right)/2\approx 1.264$ nm (g≈3.2 nm), a difference begins to appear. Note that the redshift is more enhanced in the molecule‐based systems than in the PD‐based ones across all the modeling approaches, LRA, TFHT, and QHT. This suggests that the effect of the molecule's spatial extension emerges as early as g≈3.2 nm and influences all the modeling approaches of the plasmonic system.

The inconsistency in spectral peak locations between the two systems complicates the direct comparison of their respective emission maxima. Therefore, in Figure 7A, we display averaged total emission rates, $\langle \gamma_{\mathrm{tot}}\rangle$, of the three modes, labeled as 1, 2, and 3 in Figure 6. The averaging is done over a fixed spectral window of $\hbar \omega_{f}=0.088$ eV around the spectral location of each mode. This window is chosen as it selects an area that lies within the spectral width of all the modes considered.

**FIGURE 7:**
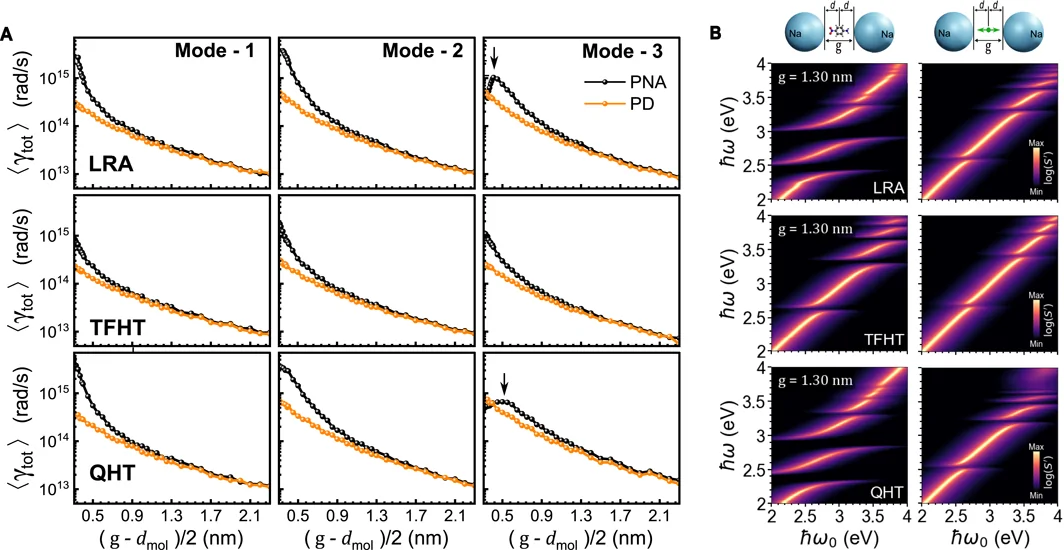
(A) The dimer‐gap‐dependent total emission rate, $\gamma_{\mathrm{tot}}$, of emitter–NaNP–dimer systems, averaged over a fixed spectral window. The first, second, and third columns (left to right) correspond respectively to the modes labeled as 1, 2, and 3 in Figure 6, while the top, middle, and bottom rows respectively correspond to the LRA, TFHT, and QHT calculation methods. The black connected symbols represent the molecule–dimer system, whereas the orange ones correspond to the PD‐dimer system. (B) Dipole spectra, $S^{\prime }$, of the molecule‐based (the left column) and PD‐based systems (the right column) as a function of the dipole frequency, $\hbar \omega_{0}$, evaluated at g=1.3 nm [$\left(g-d_{\text{mol}}\right)/2=0.314$ nm]. The emitter‐dimer configuration corresponding to each column is illustrated at the top of the respective column. The $S^{\prime }$ maps are shown for the three approaches used to describe the plasmonic systems: LRA (top row), TFHT (middle row), and QHT (bottom row). The 1, 2, and 3 labeled horizontal dashed lines in each panel indicate the spectral location of the first three modes of the system.

In alignment with what has been discussed above about the g‐dependent spectral peak positions, results of the $\langle \gamma_{\mathrm{tot}}\rangle$ seem to relatively coincide with one another at larger dimer‐gaps until they reach $\left(g-d_{\text{mol}}\right)/2\approx 1.264$ nm (g≈3.2 nm). Beyond, a visible discrepancy begins to emerge across all the modeling approaches, LRA, TFHT, and QHT, again affirming that this is where the influence of the molecule's extent begins.

It is worth noting that the metal–molecule separation at which the emitter's spatial extent begins to influence, $\left(g-d_{\text{mol}}\right)/2\approx 1.264$ nm, is consistent across all the plasmonic descriptions considered (LRA, TFHT, and QHT). This value closely matches the sub‐nanometer range, $d-d_{0}≲1$ nm, identified earlier using the LRA framework for a single AuNP (Figure 3 in Section 2.4). Interestingly, when the molecule‐metal separation is defined with respect to the ellipsoid representing the emitter's transition charge distribution, the onset of emitter‐size effects coincides with the PD result, $\left(g-d_{\text{ell}}\right)/2\approx 1$ nm, where $d_{\text{ell}}=1.2$ nm (See Figure 1). This agreement suggests that the spatial extent of a molecule universally influences emitter–plasmon interactions once the emitter‐metal separation falls below ≈1 nm, despite differences in plasmonic geometry (single NP vs. dimer), material (gold vs. sodium), and theoretical description (LRA, TFHT, and QHT).

At such small separations (in strongly confined plasmonic gaps), the scattered field varies significantly over nanometer length scales. In PD‐based approaches, however, the emitter interacts with the electromagnetic field only at a single spatial point, so the coupling is determined solely by the local field value. In contrast, a real molecule is spatially extended, such that different parts of the molecule experience different field amplitudes and directions. Consequently, the light–matter interaction is governed by the spatial overlap between the molecule and the inhomogeneous plasmonic field [20]. The resulting discrepancy between the PD‐ and PNA‐based systems therefore arises from the combination of finite molecular size and strongly confined plasmonic near fields, consistently with the physical picture described in Refs. [18, 20], as also demonstrated in the benchmark of this work in Figure 3 (Section 2.4). Hence, as stated above, the relevant quantity here is the metal–emitter separation, rather than the dimer gap size itself.

The $\langle \gamma_{\mathrm{tot}}\rangle$ corresponding to the molecule‐based system (connected black symbols) appears to dominate those corresponding to the PD‐based ones (connected orange symbols). However, as a behavior specific to the LRA‐ and QHT‐treated molecule‐based systems, the higher‐order modes considered here, particularly mode‐3, demonstrate non‐monotonic behavior, with $\langle \gamma_{\mathrm{tot}}\rangle$ displaying both rising and quenching characteristics. The rising part peaks at around $\left(g-d_{\text{mol}}\right)/2\approx 0.484$ nm (g≈1.64 nm), this is smaller for the LRA, which peaks at $\left(g-d_{\text{mol}}\right)/2\approx 0.364$ nm (g≈1.4 nm) (see the arrows in the top [LRA] and bottom [QHT] panels of the rightmost column of Figure 7A). Although rather subtle, this non‐monotonicity is also present in mode‐2, see the bottom panel (QHT) of the middle column. These higher‐order modes are quenched because they are preferentially prone to interaction with the plasmonic near‐field.

For the molecule‐based system treated within the LRA framework, the quenching part can be attributed to the cancellation of the emission signals because of the spectral mode overlap, as discussed earlier in this section. On the other hand, for the QHT model, it is because of the emergence of electron tunneling. For this approach, the tunneling does not occur through the emitter; therefore, the molecule does not function as a conductive linker between the NPs forming the dimer [47]. In this context, it is worth noting that this electron tunneling‐induced quenching effect has already been reported to begin from a dimer‐gap of g≈0.5 nm for the PD‐based system [47]. Interestingly, the characteristic gap‐value for tunneling effect value we report here (g≈1.64 nm) is a little over three times larger than that of the molecule‐based system. Indeed, in the molecule‐based QHT model, the spatially extended emitter begins to interact with the tunneling electrons at a larger separation than the PD.

To eliminate the explicit dependence on molecular size, we use the metal–emitter separation as the relevant parameter instead of the gap g (see Figures 5 and 6). Using this metric, we find that the atom–metal separation for the onset of emission quenching is $\left(g-d_{\text{mol}}\right)/2\approx 0.484$ nm. When the ellipsoidal effective charge distribution employed in the model is considered instead, the corresponding ellipsoid‐metal separation is $\left(g-d_{\text{ell}}\right)/2\approx 0.22$ nm, where $d_{\text{ell}}=1.2$ nm (See Figure 1). This value is close to the emitter–metal separation for the PD‐based model, g/2≈0.25 nm. The close agreement between these effective distances indicates that quenching is governed by the proximity of the emitter's transition charge distribution to the metal's tunneling electrons rather than by the nuclear geometry.

This behavior is illustrated schematically by overlaying the PNA molecular structure and an ellipsoid representing the emitter's transition charge distribution onto the equilibrium charge density $n_{0}$ of the QHT‐treated dimer system in Supporting Information S1: Note S3 (Supporting Information S1: Figure S3). The ellipsoid begins to overlap with the tail of $n_{0}$ at approximately the metal‐emitter separation indicated by the arrow for the QHT‐treated mode‐3 in Figure 7A, reflecting that the emitter has entered the region where metal electrons begin to spill out. The molecule remains electronically isolated in the present QHT model. Hence, this overlap does not imply charge transfer through the molecule. Instead, the observed quenching arises from non‐classical interactions between tunneling electrons and the spatially extended emitter, enhancing higher‐order plasmon modes and channeling energy into nonradiative pathways, whereas explicit conduction or molecule‐metal charge transfer is beyond the scope of this work.

Although the effective gap g for tunneling‐induced quenching differs by nearly a factor of three between the molecular and PD systems, the corresponding emitter‐metal separations are nearly identical when the ellipsoid (emitter's transition charge distribution) is the reference for the extended molecule. Hence, the separation between the metal and the emitter's transition charge distribution can serve as a universal parameter for setting the onset of tunneling‐induced quenching in emitter–dimer interactions, provided the same gap environment is used (air in our case). For different gap materials, variations in the electron affinity lead to different tunneling barriers and, consequently, different optimal gaps for the beginning of the tunneling‐induced quenching [66], limiting the universality of the metal–emitter separation parameter.

Following the above discussions, we would like to emphasize that within our QHT framework, the molecule does not act as a conductive pathway for electron tunneling between the metallic NPs. Electron tunneling occurs directly between the metal surfaces, and the molecule influences the system only through its spatial extent: by occupying the gap, it reduces the effective metal–emitter separation compared to a PD, while slightly increasing the effective metal–metal separation. Consequently, the observed shift in the different optimal gaps for the beginning of the tunneling‐induced quenching should be understood as an effective gap modification due to the finite molecular size, rather than tunneling through the molecule itself.

In Figure 7B, we explore the coupling strength of the LRA (the top row), TFHT (the middle row), and QHT (the bottom row) approaches. For this, color maps of the dipole spectra are plotted against the dipole frequency, $\hbar \omega_{0}$. The left and right columns correspond to the molecule–dimer and PD–dimer systems, respectively, with the configurations illustrated at the top of each column.

The $S^{\prime }$ maps exhibit multiple Rabi splittings corresponding to the multiple emission peaks in the spectra of the emitter–dimer systems considered here. Let us once again focus on the first three modes, which are indicated by the horizontal dashed lines in each panel and labeled as modes 1, 2, and 3 in Figure 7B. Overall, the Rabi splitting observed in the spectra of the molecule‐based systems exceeds that in the corresponding spectra of the PD‐based systems, emphasizing the impact of the molecule's spatial extent.

Let us now compare the molecule‐based systems among themselves, the first column in Figure 7B. The LRA‐ and QHT‐treated systems have shown stronger coupling, indicated by the wide Rabi splitting, than the TFHT‐based system, especially for the first two modes, 1 and 2. As can be seen from the first two columns of Figure 7A, these modes demonstrate larger total emission rates, $\langle \gamma_{\mathrm{tot}}\rangle$, than the TFHT; compare y‐axes of the top and bottom panels with the middle one for the black connected symbols. Conversely, the coupling is robust for the third mode in the TFHT‐treated system than in the LRA‐ and QHT‐treated ones. This is particularly because the gap value being analyzed here, g=1.3 nm [$\left(g-d_{\text{mol}}\right)/2=0.314$ nm], lies within the quenching regime of the g‐dependent $\langle \gamma_{\mathrm{tot}}\rangle$ curves for mode‐3 in both the LRA‐ and QHT‐treated systems (see the arrows in the rightmost column of Figure 7A).

Although we focus here on a PNA molecule, similar trends are expected for larger or smaller molecules, as well as for more isotropic or anisotropic emitters. The onset gap for size effects and the magnitude of the Rabi splitting depend on the molecular size and transition dipole moment, with smaller or weaker emitters requiring stronger field confinement. This is supported by Refs. [18, 20, 67], which, within the LRA framework, show that methylene blue (MB), zinc phthalocyanine (ZnPc), and Chromophores, despite their different sizes and transition dipole characters, exhibit clear deviations from PD descriptions at sub‐nanometer emitter–plasmon separations. As shown here, the same behavior persists across different plasmonic models (LRA, TFHT, and QHT), where consistent deviations between PNA‐ and PD‐based descriptions are observed at the same emitter–plasmon separation range. Therefore, while the quantitative results are molecule‐specific, the qualitative conclusions are broadly applicable, reflecting the general interaction between spatially extended molecules and strongly inhomogeneous plasmonic fields.

By explicitly accounting for both the emitter's spatial extent and plasmonic quantum effects, the DFT–QHT framework provides a physical interpretation of non‐classical emitter–plasmon interactions at sub‐nanometer separations. Such plasmonic gaps have been experimentally realized in noble‐metal systems using nanoparticle‐on‐mirror (NPoM) geometries [17], collapsible nanofinger techniques [66, 68], atomic force microscopy (AFM) [15], and scanning tunneling microscopy (STM) junctions [69]. In these platforms, non‐monotonic gap‐dependent emission, tunneling‐induced fluorescence, and Raman quenching [66], as well as gap‐dependent variations in coupling strength [69], have been observed. The present approach can be readily adapted to the plasmonic materials employed in these experiments and used to interpret observations in regimes where PD models break down.

## 3 Conclusions

In this work, we have presented an efficient QHT–DFT hybrid approach that enables consideration of both the emitter's spatial extent and plasmonic quantum effects in modeling emitter‐plasmon interactions. For this, a physical molecule is calculated using TDDFT, and the plasmonic system is modeled using QHT. Using LRA, we first benchmark this physically extended molecule against a PD, whose moment is estimated from the transition charge density of the molecule itself, where consistent emission properties are observed at large emitter–plasmon separations. Furthermore, consistent with reports in the literature, the impact of physical extent becomes apparent within the sub‐nanometer separation range, leading to differences between the molecule‐ and PD‐based systems in both emission enhancement and coupling strength. Thereafter, the combined effects of the quantum and the emitter's spatial extent are examined by progressively introducing quantum phenomena into a plasmonic dimer, starting from the fully classical LRA, moving to the nonlocal TFHT, and ultimately reaching the full QHT.

The spatial extent effect begins at a dimer gap size, g, of about g≈3.2 nm across all the LRA, TFHT, and QHT models, resulting in a difference in the emission rate strength as well as spectral peak positions. Beyond this gap, a good agreement is observed between the molecule‐ and PD‐based systems for all the models (LRA, TFHT, and QHT). Interestingly, the maxima of the highest order mode analyzed in this work exhibit a non‐monotonic dependence on the dimer gap, characterized by increasing and decreasing parts, specifically in the LRA and QHT approaches. In the latter case, the quenching occurs due to tunneling electrons that start to emerge after a gap size of g≈1.64 nm. More remarkably, this gap is found to be approximately three times larger in the spatially extended molecule than in the PD when both are coupled to a QHT‐treated system. In the LRA, the quenching occurs as a result of modal overlap due to the interaction between the extended molecule and the LRA‐induced strong field at its location. The gap size at which the modal‐overlap‐induced quenching begins to emerge is less than the electron‐tunneling‐induced one and is demarcated by a spectral overlap‐dominated zone in the spectral‐ and gap‐dependent map of the emission properties.

Rather than the gap itself, the metal–emitter separation emerges as a universal parameter for analyzing emitter–plasmon interactions. The onset of emitter‐size effects consistently occurs at ≈1 nm across all theoretical descriptions considered (LRA, TFHT, and QHT), demonstrating a universal length scale governed by the proximity of the emitter's effective charge distribution to the metal rather than its atoms. Apparent differences between PD‐ and molecule‐based models primarily arise from the effective reduction of the metal‐emitter separation caused by finite molecular size. For tunneling‐induced quenching, gap values of ≈0.22 nm (measured from the emitter's effective charge distribution) for the PNA‐dimer system (this work) and ≈0.25 nm for the PD‐dimer system [47] are in close agreement, further supporting the metal‐emitter separation as a universal descriptor for the onset of quenching, provided the same gap material is considered.

Further, the spatial extent of the emitter is observed to influence the plasmon–emitter coupling strength, as evidenced by the significantly larger Rabi splittings in the dipole spectra of the molecule–dimer system compared to the PD‐dimer system across all models, LRA, TFHT, and QHT. The results also demonstrate that the coupling strength of the highest‐order mode considered here is more robust in the TFHT‐treated molecule‐dimer system compared to those treated with QHT and LRA, as the corresponding modes in the latter cases are subject to quenching induced by quantum tunneling and mode overlap, respectively. By contrast, the coupling strengths associated with the lower‐order modes, which are not affected by quenching, exhibit larger Rabi splittings in the dipole spectra of the QHT‐ and LRA‐treated molecule‐dimer systems compared to those of the TFHT‐treated system.

The results, specifically, the discrepancies in emission rate properties and coupling strengths between the molecule‐ and PD‐based systems, underscore that both the plasmonic quantum effects and spatial extent of the emitter are critical factors for accurately predicting emitter‐plasmon responses in narrow plasmonic gaps. These findings firmly establish the QHT–DFT hybrid model's capability to capture these effects effectively. Hence, this model could be an asset to address the difficulty often faced in reproducing experimental emitter–plasmon optical responses using PD‐based systems, which often necessitate using the dipole moment of the PD as a tuning parameter.

## 4 Methods

We employed the 2.5D axis‐symmetric modeling approach to calculate emission rates of the emitters near the plasmonic systems. This requires that all the vector fields involved in the simulation be defined in cylindrical coordinate system, ν(ρ,ϕ,z), and written in terms of angular harmonics, m∈Z as: $\nu \left(\rho ,\phi ,z\right)=\sum_{m}\nu^{\left(m\right)}\left(\rho ,z\right)e^{\left(-im\phi \right)}$. This helps to significantly reduces computational demands in terms of memory usage and storage requirements, as the originally three‐dimensional problems (Equations (2a), (2b), and 5) can be reduced to a small set of two‐dimensional problems, specifically $2m_{\text{max}}+1$ in total, where $m_{\text{max}}$ denotes the highest angular mode considered. For emitters placed exactly on the symmetry axis and for sub‐wavelength structures, as is the case here, a cutoff of $2m_{\max}<3$ is typically sufficient to capture the system's behavior accurately. In this section, we present and discuss the explicit expressions of the 2.5D models used in this work.

### 4.1 QHT in 2.5D

We solve the system of QHT equation (Equations 2a and 2b) in a commercially available FEM software (COMSOL Multiphysics) [58]. For this, weak form of the differential equations can be obtained by multiplying them by their respective test functions, $\tilde{E}$ for Equation (2a) and $\tilde{P}$ for Equation (2b), and applying the standard variational procedure, which involves integration by parts, the assumption that the boundary integral vanishes, and azimuthal dependence [42, 53]. The electric field vector E and the polarization vector P are expressed with an angular dependence of the form $e^{-im\phi }$, while their corresponding test functions take the form $e^{im\phi }$. This choice causes the angular dependence to cancel out upon integration over the azimuthal coordinate, leaving only equations that depend on ρ and z. As a result, the simulation can be performed in the ρ‐z plane. However, since the first‐order energy functional term, ${\left(\frac{\delta G}{\delta n}\right)}_{1}$ in Equation (2b) involves second‐order derivatives, we introduce an auxiliary variable $F=\nabla n_{1}$ with $n_{1}=\left(1/e\right)\nabla \cdot P$. This reformulation allows us to express $\nabla^{2}n_{1}$ as ∇⋅F, thereby reducing the problem to involve only first‐order derivatives.

The resulting system of equations used to compute the unknowns E, P, and F is given by [42, 47, 48]:

(10a) $$\begin{array}{ll} 2\pi \int & \left\{\left(\nabla \times E^{\left(m\right)}\right)\cdot \left(\nabla \times {\tilde{E}}^{\left(m\right)}\right)\right.\\ & \;\left.-\left(\frac{\omega^{2}}{c^{2}}E^{\left(m\right)}+\mu_{0}\omega^{2}p^{\left(m\right)}\right)\cdot {\tilde{E}}^{\left(m\right)}\right\}\rho \;d\rho \;dz=0, \end{array}$$

(10b) $$\begin{array}{ll} 2\pi \int & \left\{-\frac{e}{m_{e}}{\left(\frac{\delta G\left[n\right]}{\delta n}\right)}_{1}^{\left(m\right)}\left(\nabla \cdot {\tilde{p}}^{\left(m\right)}\right)\right.\\ & \;+\frac{1}{n_{0}}\left[\left(\omega^{2}+i\gamma \omega \right)p^{\left(m\right)}\right.\\ & \;\left.\left.+\epsilon_{0}\omega_{p}^{2}E^{\left(m\right)}\right]\cdot {\tilde{p}}^{\left(m\right)}\right\}\rho \;d\rho \;dz=0, \end{array}$$

(10c) $$\begin{array}{ll} 2\pi \int & \left\{\left(\nabla \cdot p^{\left(m\right)}\right)\left(\nabla \cdot {\tilde{F}}^{\left(m\right)}\right)\right.\\ & \;\left.+\epsilon F^{\left(m\right)}\cdot {\tilde{F}}^{\left(m\right)}\right\}\rho \;d\rho \;dz=0, \end{array}$$

where ∇ is a modified operator, $\nabla \to \nabla +im/\rho \;\hat{\phi }$, since the vector fields $\nu^{\left(m\right)}$ depend only on ρ and z.

### 4.2 The PNA Molecule Field in 2.5D

The ellipsoidal charge distribution of the PNA molecule is well suited for 2.5D axisymmetric modeling. By aligning the symmetry axis of the prolate ellipsoid along the z‐axis in a cylindrical coordinate system (ρ,ϕ,z), the electric field can be assumed azimuthally symmetric, effectively eliminating any dependence on the azimuthal angle ϕ. As a result, only the radial $\left(E_{\rho }\right)$ and axial $\left(E_{z}\right)$ components of the electric field are relevant for the 2.5D formulation.

Although the ellipsoidal geometry is axis‐symmetric, the charge distribution on its surface is not (see Figure 2A); that is, charges with the same z and ρ coordinates can have different values. Hence, the proper method for establishing the fields suitable for the 2.5D method, $E_{\rho }$ and $E_{z}$, is to compute the azimuthally averaged electric field of the 3D field (Equation 5) after expressing it in cylindrical coordinates. This was accomplished by averaging the electric field values within each bin after discretising the ρz space into bins. However, this approach was observed to reduce spatial resolution because of the binning, although it maintains the overall accuracy of the electrostatic field, producing quantitative results that are consistent with the full 3D simulation.

On the other hand, a straightforward projection of the x and y components of the 3D Cartesian electric field from Equation (5) onto the ρ direction has provided satisfactory results both in spatial resolution as well as field value. This success is predominantly because of the polarization of the molecule, which is z‐dominated, making the contribution from the azimuthal field significantly small (see Figure 2). We therefore apply this projection method to calculate the ρ‐component of the electric field for the implementation of the axis‐symmetric (2.5D) modeling framework.

The radial component, $E_{\rho }$ at the observation point r is obtained by projecting the Cartesian components $E_{x}$ and $E_{y}$ (see Equation 5) onto the fixed local radial unit vector defined at r: $e_{\rho }=\left[xe_{x}+ye_{y}\right]/\rho$ with $\rho =\sqrt{x^{2}+y^{2}}$. Therefore, the radial component of the field at position r, due to all source charges $q_{j}$, is given by $E_{\rho }=\left(E_{x}e_{x}+E_{y}e_{y}\right)\cdot e_{\rho }$,

(11) $$E_{\rho }=\sum_{j}\left(\frac{q_{j}}{4\pi \varepsilon_{0}|r-r_{j}{|}^{3}}\cdot \frac{\left(x-x_{j}\right)x+\left(y-y_{j}\right)y}{\rho }\right).$$

The components $E_{\rho }$ and $E_{z}$ then serve as input parameters for the source term in the wave equation, Equation (10a), and are defined on boundary of an elongated ellipse, measuring 0.6 nm along the semimajor axis and 0.4 nm along the semiminor axis, that represents the physical PNA molecule in the 2.5D simulations.

## Author Contributions

All authors have accepted responsibility for the entire content of this manuscript and consented to its submission to the journal, reviewed all the results and approved the final version of the manuscript. The original idea was conceived by C.C., F.D.S., and S.C. Numerical simulations were performed by T.O.O., with input from C.C., and the DFT simulation was carried out by G.G., with input from S.C. G.Á.‐P., H.H., and C.C. contributed to the discussion. Data were analyzed by T.O.O., with input from C.C. Figures were prepared, and the first draft of the manuscript was written by T.O.O. The manuscript was subsequently revised with inputs from C.C., G.G., G.Á.‐P., F.P., and F.D.S.

## Conflicts of Interest

The authors declare no conflicts of interest.

## Supporting information


Supporting Information S1

## Data Availability

The data that support the findings of this study are available from the corresponding author upon reasonable request.
