# Supplementary material for: Plasmonic‐Quantum and Emitter's Finite‐Size Effects in Plasmon‐Emitter Coupling: A QHT–DFT Hybrid Approach
Source: Nanophotonics. 2026 Mar 16;15(6):e70044. doi: 10.1002/nap2.70044 (PMC13642538; doi:10.1002/nap2.70044)
Supplement: Supplementary file 1 [file NAP2-15-e70044-s001.pdf]

SUPPLEMENTARY INFORMATION for Plasmonic-quantum and emitter's finite-size effects in plasmon-emitter coupling: a QHT-DFT hybrid approach

Tadele Orbula Otomalo,[1] Gabriel Gil,[2] Gonzalo Álvarez-Pérez,[1] Huatian Hu,[1]
Ferruccio Pisanello,[1] Stefano Corni,[3] Fabio Della Sala,[4,1] and Cristian Ciracì[5,1]

[1]*Istituto Italiano di Tecnologia, Center for Biomolecular Nanotechnologies, Via Barsanti 14, 73010 Arnesano, Italy*
[2]*Università del Piemonte Orientale, Alessandria, Italy*
[3]*Università degli Studi di Padova, Padova, Italy*
[4]*Institute for Microelectronics and Microsystems (CNR-IMM), Via Monteroni, Campus Unisalento, 73100 Lecce, Italy*
[5]*Neurophos Inc., 4120 Freidrich Ln, Austin, TX 78744, United States*

SUPPLEMENTARY NOTE 1. CALCULATING THE OPTICAL RESPONSES FROM THE EM FIELDS

An excited emitter can relax either radiatively or non-radiatively, and the corresponding emission rates are of particular interest, as the surrounding environment, specifically, the plasmonic system in this context, can significantly influence them [1]. The decay rates arising from the emitter-plasmon interactions studied here are evaluated from the computed EM fields. The non-radiative decay rate, in particular, is obtained by integrating the power absorbed within volume V of the plasmonic system, as follows [1]:

$$\gamma_{nr} = \frac{1}{2}\frac{\gamma_0}{W_0}\int \mathrm{Re}\left\{\mathbf{J}^* \cdot \mathbf{E}\right\} dV \tag{SI-1}$$

Here, $\gamma_0 = \frac{\omega^3|\mathbf{P}|^2}{3\hbar\pi\varepsilon_0 c^3}$ and $W_0 = \frac{\omega^4|\mathbf{P}|^2}{12\pi\varepsilon_0 c^3}$ represent radiative decay rate and total power radiated by a dipole in free space, respectively.

The radiative decay rate of the system can be evaluated by integrating the power flowing out of the simulation domain Ω through its boundary surface S, as [1]:

$$\gamma_r = \frac{1}{2}\frac{\gamma_0}{W_0}\int_{\partial\Omega} \mathrm{Re}\left\{\mathbf{E}\times\mathbf{H}^*\right\} dS. \tag{SI-2}$$

The total emission rate can then be evaluated as the sum of these contributions, $\gamma_{\mathrm{tot}} = \gamma_r + \gamma_{nr}$. Accordingly, the emission efficiency (or quantum yield) of the system can be defined as $q(\omega) = \gamma_r/\gamma_{\mathrm{tot}}$.

The Lamb shift can be directly related to the total decay rate through the system's Green's function at the emitter location $\mathbf{r}_0$ as [1–6]:

$$\delta\omega(\mathbf{r}_0,\omega) = \frac{1}{\pi\hbar\varepsilon_0 c^2}\mathcal{P}\int_0^\infty \frac{\omega'^2}{\omega'-\omega}|\boldsymbol{\mu}|^2\left[\hat{\mathbf{n}}_\mu\cdot\mathrm{Im}\left\{\mathbf{G}(\mathbf{r}_0,\mathbf{r}_0;\omega')\right\}\cdot\hat{\mathbf{n}}_\mu\right]d\omega'. \tag{SI-3}$$

Here, $\mathcal{P}$ denotes the Cauchy principal value, $\mathbf{G}$ is Green's function of the system and $\hat{\mathbf{n}}_\mu$ is a unit vector in the direction of the moment, $\boldsymbol{\mu}$.

The total emission rate for the same system can also be expressed in terms of $\mathbf{G}$ through LDOS, $\rho(\mathbf{r}_0,\omega) = 6\omega/(\pi c^2)\left[\hat{\mathbf{n}}_\mu\cdot\mathrm{Im}\left\{\mathbf{G}(\mathbf{r}_0,\mathbf{r}_0;\omega)\right.\right.$ using Fermi's golden rule [3, 7–9]:

$$\gamma_{\mathrm{tot}}(\mathbf{r}_0,\omega) = \frac{\pi\omega}{3\hbar\varepsilon_0}|\boldsymbol{\mu}|^2\rho(\mathbf{r}_0,\omega). \tag{SI-4}$$

Substituting Eq. SI-3 and simplifying yields:

$$\delta\omega(\mathbf{r}_0,\omega) = \frac{1}{2\pi}\mathcal{P}\int_0^\infty \frac{\gamma_{\mathrm{tot}}(\mathbf{r}_0,\omega')}{\omega'-\omega}d\omega'. \tag{SI-5}$$

This expression is then treated as a Kramers-Kronig relation by extending the lower integration bound to $-\infty$ [4], and is subsequently solved to determine the Lamb shift.

SUPPLEMENTARY NOTE 2. EMISSION PROPERTIES OF PNA AND PD NEAR PLASMONIC SYSTEMS

In **Supplementary Fig. 1**, we plot the spectral-dependent emission properties of PNA- and PD-based systems for different d in the sub-nanometer range. Fig. **Supplementary Fig. 1**(B) displays the non-radiative, $\gamma_{\rm nr}$, (right panel) and radiative, $\gamma_{\rm r}$, (left panel) emission rates of the molecule-AuNP system as a function of the emission frequency, $\hbar\omega$. In addition to the aforementioned enhancement of the emission rates, we also observe a slight red shift of the resonance peaks with decreasing d. This redshift is indicative of a strong coupling between the molecule and the AuNP. As the separation narrows, interaction between the molecule's dipole field and the plasmonic mode leads to the formation of a bonding-like hybrid mode with reduced photon energy [10]. Such interactions often result in an increased LDOS, placing the molecule in a regime of reversible energy exchange with the AuNP, also known as coherent Rabi oscillation. The signature induced by such oscillation can be observed as the so-called photonic Rabi splitting in the spectral response of the coupled system [3, 11].

However, when a PD replaces the physical molecule [see **Supplementary Fig. 1**(C) and (D)], the previously observed features with decreasing d, namely the emission rate enhancement, redshift, and strong coupling [see Sec. II(C) in the main text], are significantly suppressed or negligible. As shown in **Supplementary Fig. 1**(D), the enhancement and redshift are barely noticeable as d decreases.

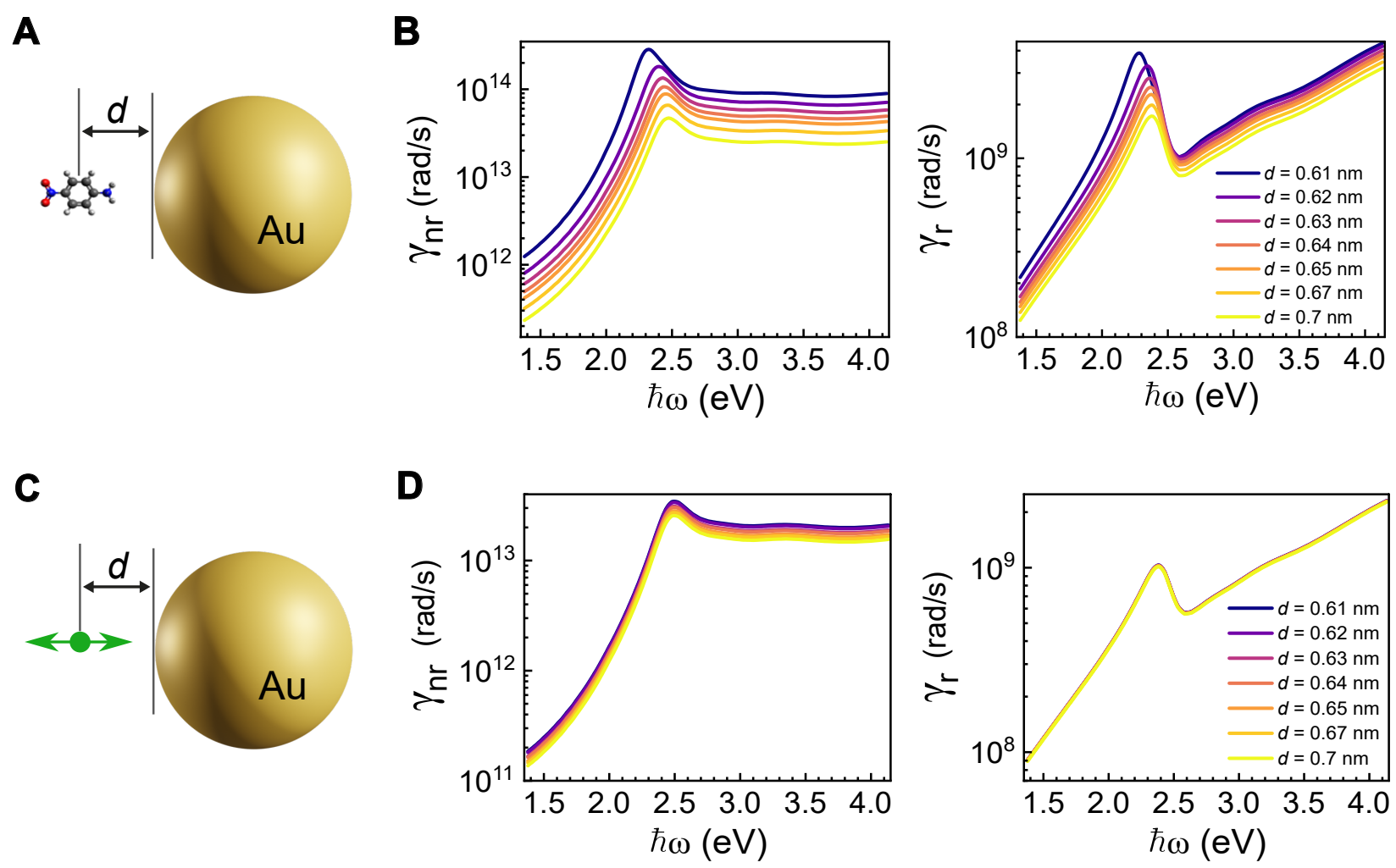

Supplementary Fig. 1: Comparison of emission properties of PNA molecule and PD near a single gold NP (AuNP, 40 nm in diameter) at varying emitter-AuNP separation distances, d. (A) and (B) correspond to the molecule-AuNP interaction: (A) Schematic illustration of the molecule-AuNP system. The separation distance d is measured from the geometric center of the molecule to the surface of the AuNP. (B) non-radiative (γ_{nr}, left panel) and radiative (γ_r, right panel) emission rates, in log scale, as functions of photon energy, $\hbar\omega$, for different values of d ($0.61 \le d \le 0.70$ nm). (B) and (C) correspond to the PD-AuNP interaction: (B) Schematic of the PD-AuNP system. Here, d is defined as the distance from the PD location to the AuNP surface. (C) Same as (B), but for a PD-AuNP system.

Supplementary Fig. 2 displays the spectral- and g-dependent non-radiative and radiative emission rates of the emitter-dimer system. The spectra are depicted in **Supplementary Fig. 2**(B) and (D), for the molecule- and PD-based systems, respectively. As expected, the overall emission rates are significantly enhanced compared to those in **Supplementary Fig. 1**(B) and (D) for the emitter-AuNP systems. The bonding and antibonding modes [12, 13], created by the interaction of LSPR modes of the AuNPs, are distinctly visible in the spectrum of $\gamma_{\rm nr}$. The bonding dipolar plasmon (BDP) mode for the molecule-dimer system [left panel in **Supplementary Fig. 2**(B)] is observed to redshift and increase dramatically with decreasing g, eventually becoming the dominant mode in the spectrum. In contrast, the PD-based system exhibits a BDP mode as a shoulder on the red side of its LSPR peak [left panel in **Supplementary Fig. 2**(D)], and its overall optical response remains largely insensitive to the decreasing gap size [**Supplementary Fig. 2**(D)]. Qualitatively, these spectral response trends are similar to those observed earlier for the monomer-based systems in **Supplementary Fig. 1**.

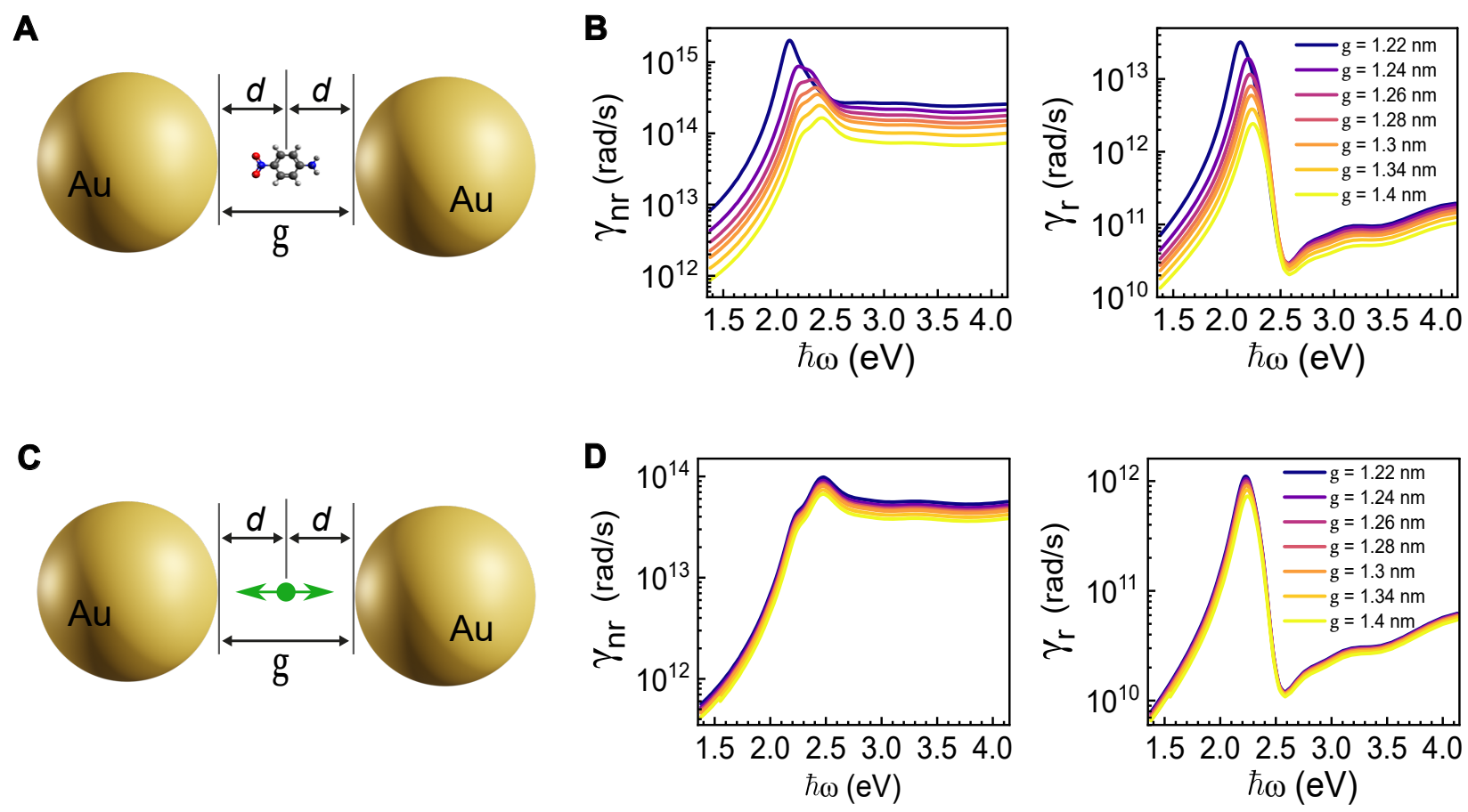

Supplementary Fig. 2: Comparison of emission properties of PNA molecule and PD, both placed in the middle of a plasmonic dimer-gap. The dimer consists of two identical AuNPs with diameters of 40 nm. The gap width is defined as $g = 2d$, where d is the distance from the surface of either AuNP to the gap center. (A) and (B) correspond to the molecule-dimer interaction: (A) Schematic illustration of the dimer-molecule system. (B) non-radiative (γ_{nr}, left panel) and radiative (γ_r, right panel) emission rates, in log scale, as functions of photon energy, $\hbar\omega$, for different dimer-gap, g ($1.22 \leq g \leq 1.4$ nm). (C) and (D) correspond to the PD-dimer interaction: (C) Schematic of the PD-dimer system. In this case, d is the distance from the dipole location, centered in the dimer-gap, to the surface of either AuNP. (D) Same as (B), but for a PD-based system.

SUPPLEMENTARY NOTE 3. EQUILIBRIUM ELECTRON DENSITY, n_0

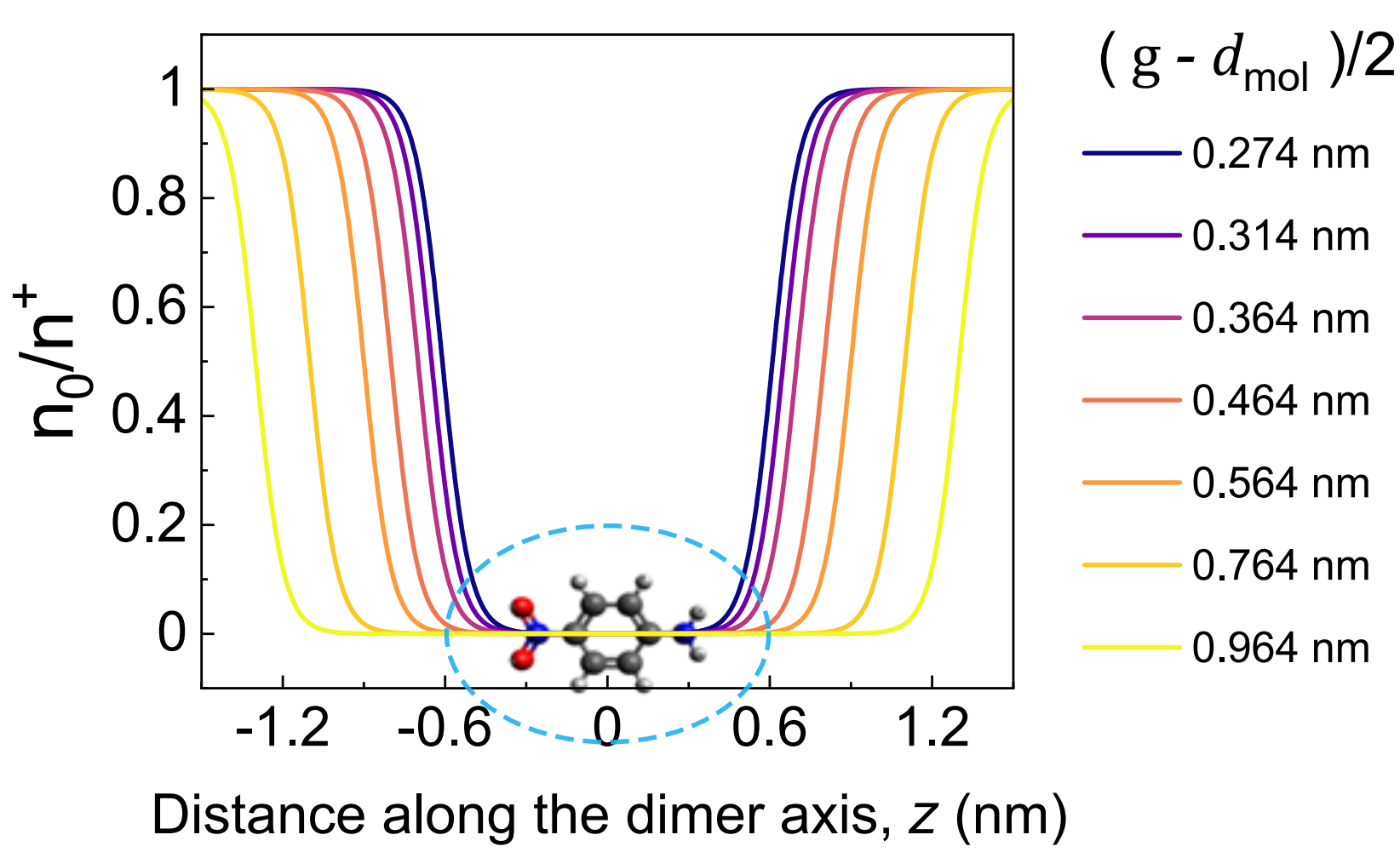

Supplementary Fig. 3: The spatially dependent equilibrium electron density, n_0, extracted along the dimer axis, for different dimer-gaps or emitter-metal separations, $(g - d_{mol})/2 = 0.274$ nm to 0.964 nm, and normalized by the background charge density value n^+. The schematic insertion of the molecular structure of PNA illustrates its spatial extent (from -0.336 nm to 0.336 nm) within the dimer gap along the dimer axis, while the dashed ellipse represents the surface of the emitter's transition charge distribution, extending from -0.6 nm to 0.6 nm along the dimer axis.

SUPPLEMENTARY REFERENCES

[1] L. Novotny and B. Hecht, *Principles of nano-optics* (Cambridge University Press, 2012).
[2] Y. Zhang, Z.-C. Dong, and J. Aizpurua, The Journal of Physical Chemistry C **124**, 4674 (2020).
[3] C. Ciracı, R. Jurga, M. Khalid, and F. Della Sala, Nanophotonics **8**, 1821 (2019).
[4] S. D'Agostino, F. Alpeggiani, and L. C. Andreani, Optics Express **21**, 27602 (2013).
[5] P. Yao, C. Van Vlack, A. Reza, M. Patterson, M. M. Dignam, and S. Hughes, Physical Review B—Condensed Matter and Materials Physics **80**, 195106 (2009).
[6] H. T. Dung, L. Knöll, and D.-G. Welsch, Physical Review A **62**, 053804 (2000).
[7] P. Anger, P. Bharadwaj, and L. Novotny, Physical Review Letters **96**, 113002 (2006).
[8] C. Ciracı, A. Rose, C. Argyropoulos, and D. R. Smith, JOSA B **31**, 2601 (2014).
[9] H. M. Baghramyan and C. Ciracı, Nanophotonics **11**, 2473 (2022).
[10] F. Bloksma and P. Zijlstra, The Journal of Physical Chemistry C **125**, 22084 (2021).
[11] G. Zengin, M. Wersäll, S. Nilsson, T. J. Antosiewicz, M. Käll, and T. Shegai, Physical Review Letters **114**, 157401 (2015).
[12] H. Wang, Scientific Reports **8**, 9589 (2018).
[13] L. Yang, H. Wang, Y. Fang, and Z. Li, ACS Nano **10**, 1580 (2016).
